# Somatostatin receptor 2 expression in nasopharyngeal cancer is induced by Epstein Barr virus infection: impact on prognosis, imaging and therapy

**DOI:** 10.1038/s41467-020-20308-8

**Published:** 2021-01-05

**Authors:** Matt Lechner, Volker H. Schartinger, Christopher D. Steele, Wen Long Nei, Marc Lucas Ooft, Liesa-Marie Schreiber, Christodoulos P. Pipinikas, Grace Tin-Yun Chung, Yuk Yu Chan, Feng Wu, Ka-Fai To, Chi Man Tsang, Wayne Pearce, Daniele Morelli, Martin Philpott, Liam Masterson, Reshma Nibhani, Graham Wells, Christopher G. Bell, Julia Koller, Susanne Delecluse, Yim Ling Yip, Jacklyn Liu, Cillian T. Forde, Martin D. Forster, Amrita Jay, József Dudás, Annika Krapp, Simon Wan, Christian Uprimny, Susanne Sprung, Johannes Haybaeck, Tim R. Fenton, Kerry Chester, Christina Thirlwell, Gary Royle, Teresa Marafioti, Rajeev Gupta, Sagung Rai Indrasari, Camelia Herdini, Mohd Afiq Mohd Slim, I. Indrawati, Liam Sutton, Renske Fles, Bing Tan, Joe Yeong, Amit Jain, Shuting Han, Haitao Wang, Kelvin S. H. Loke, Wan He, Ruilian Xu, Hongtao Jin, Zhiqiang Cheng, David Howard, Peter H. Hwang, Quynh-Thu Le, Joshua K. Tay, Robert B. West, Sai Wah Tsao, Tim Meyer, Herbert Riechelmann, Udo Oppermann, Henri-Jacques Delecluse, Stefan M. Willems, Melvin L. K. Chua, Pierre Busson, Kwok Wai Lo, Guido Wollmann, Nischalan Pillay, Bart Vanhaesebroeck, Valerie J. Lund

**Affiliations:** 1 UCL Cancer Institute, University College London, London, UK; 2 Department of Otolaryngology-Head and Neck Surgery, Stanford University School of Medicine, Palo Alto, CA USA; 3 Barts Health NHS Trust, London, UK; 4 Royal National Throat, Nose and Ear Hospital and Head and Neck Centre, University College London Hospitals NHS Trust, London, UK; 5 Department of Otorhinolaryngology, Medical University of Innsbruck, Innsbruck, Austria; 6 Divisions of Radiation Oncology and Medical Sciences, National Cancer Centre, Singapore, Singapore; 7 Oncology Academic Programme, Duke-NUS Medical School, Singapore, Singapore; 8 King´s College Hospitals, NHS Foundation Trust, London, UK; 9 Department of Pathology, University Medical Center Utrecht, Utrecht, The Netherlands; 10 Institute of Virology and Christian Doppler Laboratory for Viral Immunotherapy of Cancer, Medical University of Innsbruck, Innsbruck, Austria; 11 Department of Anatomical and Cellular Pathology and State Key Laboratory of Translational Oncology, The Chinese University of Hong Kong, Hong Kong, China; 12 Botnar Research Centre, University of Oxford, Oxford, UK; 13 Department of Otolaryngology, Addenbrooke’s Hospital, Cambridge, UK; 14 William Harvey Research Institute, Barts and The London School of Medicine and Dentistry, Queen Mary University of London, London, UK; 15 German Cancer Research Centre (DKFZ) and Inserm, Unit F100/U1074, Heidelberg, Germany; 16 School of Biomedical Sciences, Li Ka Shing Faculty of Medicine, University of Hong Kong, Hong Kong, China; 17 Department of Histopathology, University College London Hospitals NHS Trust, Euston Road, London, UK; 18 Institute of Nuclear Medicine, University College Hospital, Euston Road, London, UK; 19 Department of Nuclear Medicine, Medical University of Innsbruck, Innsbruck, Austria; 20 Department of Pathology, Neuropathology and Molecular Pathology, Medical University of Innsbruck, Innsbruck, Austria; 21 Diagnostic & Research Center for Molecular Biomedicine, Institute of Pathology, Medical University of Graz, Graz, Austria; 22 School of Biosciences, University of Kent, Canterbury, UK; 23 University of Exeter College of Medicine and Health, Exeter, UK; 24 ENT Head and Neck Surgery Department, Universitas Gadjah Mada, Dr. Sardjito Hospital, Yogyakarta, Indonesia; 25 Department of Ear, Nose and Throat, University Hospital Crosshouse, Crosshouse, Kilmarnock UK; 26 Department of Anatomical Pathology, Universitas Gadjah Mada, Dr. Sardjito Hospital, Yogyakarta, Indonesia; 27 Department of Head and Neck Surgery and Oncology, Netherlands Cancer Institute, Amsterdam, The Netherlands; 28 Department of ENT/Head and Neck Surgery, Maastricht University Medical Center (MUMC), Maastricht, The Netherlands; 29 Department of Anatomical Pathology, Singapore General Hospital, Singapore, Singapore; 30 Institute of Molecular and Cell Biology, A*STAR, Singapore, Singapore; 31 Division of Medical Oncology, National Cancer Centre, Singapore, Singapore; 32 Department of Nuclear Medicine and Molecular Imaging, Singapore General Hospital, Singapore, Singapore; 33 Department of Oncology, The Second Clinical Medical College, Shenzhen People’s Hospital, Jinan University, Shenzhen, Guangdong China; 34 Department of Pathology, The Second Clinical Medical College, Shenzhen People’s Hospital, Jinan University, Shenzhen, Guangdong China; 35 ENT Department, Charing Cross Hospital, Imperial College Healthcare NHS Trust, London, UK; 36 Department of Radiation Oncology, Stanford University School of Medicine, Palo Alto, CA USA; 37 Department of Pathology, Stanford University School of Medicine, Palo Alto, CA USA; 38 Department of Otolaryngology—Head and Neck Surgery, National University of Singapore, Singapore, Singapore; 39 Freiburg Institute for Advanced Studies (FRIAS), University of Freiburg, 79085 Freiburg, Germany; 40 Department of Pathology, University Medical Center Groningen, Groningen, The Netherlands; 41 CNRS-UMR 9018-METSY, Gustave Roussy and Université Paris-Saclay, Villejuif, France; 42 Department of Cellular and Molecular Pathology, Royal National Orthopaedic Hospital NHS Trust, Stanmore, UK

**Keywords:** Head and neck cancer, Cancer

## Abstract

Nasopharyngeal cancer (NPC), endemic in Southeast Asia, lacks effective diagnostic and therapeutic strategies. Even in high-income countries the 5-year survival rate for stage IV NPC is less than 40%. Here we report high somatostatin receptor 2 (SSTR2) expression in multiple clinical cohorts comprising 402 primary, locally recurrent and metastatic NPCs. We show that SSTR2 expression is induced by the Epstein–Barr virus (EBV) latent membrane protein 1 (LMP1) via the NF-κB pathway. Using cell-based and preclinical rodent models, we demonstrate the therapeutic potential of SSTR2 targeting using a cytotoxic drug conjugate, PEN-221, which is found to be superior to FDA-approved SSTR2-binding cytostatic agents. Furthermore, we reveal significant correlation of SSTR expression with increased rates of survival and report in vivo uptake of the SSTR2-binding ^68^Ga-DOTA-peptide radioconjugate in PET-CT scanning in a clinical trial of NPC patients (NCT03670342). These findings reveal a key role in EBV-associated NPC for SSTR2 in infection, imaging, targeted therapy and survival.

## Abstract (web summary)

Nasopharyngeal carcinoma (NPC) lacks effective diagnostic and therapeutic strategies, in particular at advanced stages. Here, the authors show that expression of the somatostatin receptor 2 is induced by Epstein-Barr virus in NPC and has a key role in the diagnosis, imaging, targeted therapies and prognosis of NPC.

## Introduction

**Fig. 1:**
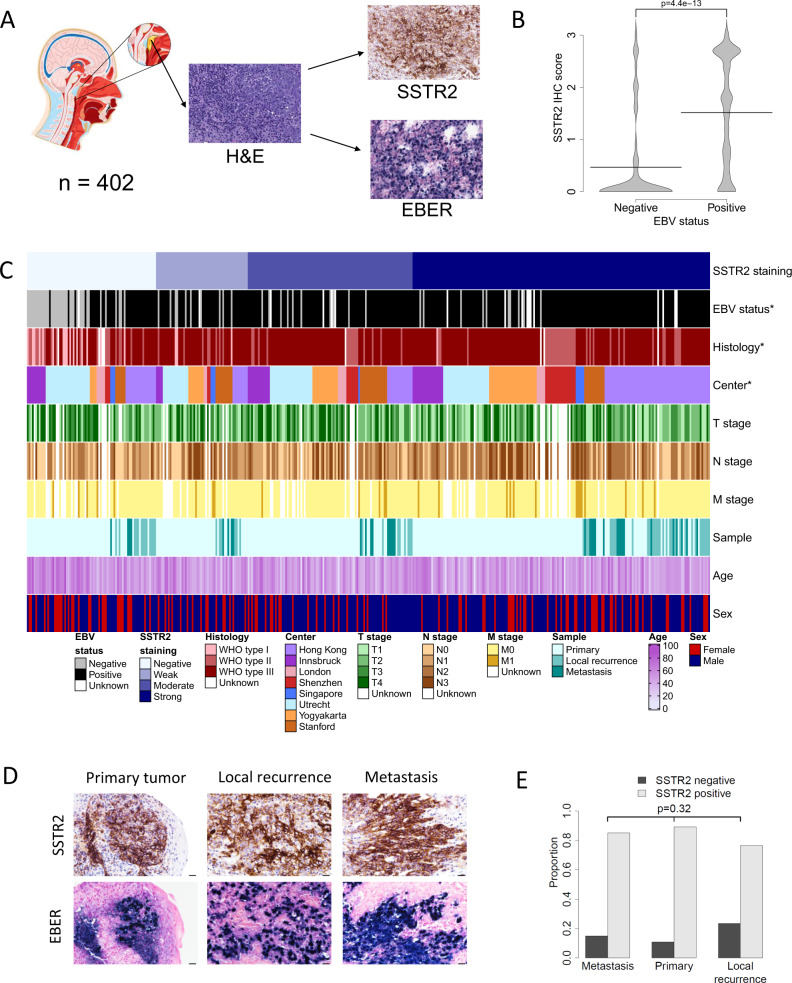
Evaluation of SSTR2 expression in a multi-institution clinical cohort of NPC. **a** Anatomical localization and representative images of hematoxylin and eosin (H&E) stained histology, somatostatin receptor 2 (SSTR2) expression assessed by immunohistochemistry (IHC) and Epstein-Barr Virus (EBV)-encoded small RNAs (EBER) assessed by in situ histochemistry). **b** Beanplot of the SSTR2 IHC score in EBV-positive (*n* = 278) and EBV negative (*n* = 60) cases of NPC (W = 3085.5, *p* = 4.0e-14, Wilcoxon two-sided test). **c** Heatmap representation of the clinical annotations in relation to SSTR2 expression levels (asterisks indicate significant associations with SSTR2 expression using multivariate analysis). **d**, **e** SSTR2 status and EBV status were not statistically different in the primary (*n* = 37), local recurrent (*n* = 47), and metastatic (*n* = 20) tumor tissue (images of rNPC42; ×400; scale bar 25 µm and summary of data; two-sided Fisher’s test on proportion of SSTR2 positivity in primary, metastatic and local recurrence samples; *p* = 0.32). Representative tumor samples from a single patient are shown in **d**. Source Data are provided as a Source data file.

NPC is a malignant epithelial tumor showing squamous differentiation^1^. It occurs most frequently in the pharyngeal recess (fossa of Rosenmuller), an area that is difficult to access surgically due to the anatomical constraints in creating open access for surgical resection (Fig. 1a). NPC is classified into three histological subtypes by the World Health Organization (WHO I–III). The non-keratinizing NPC subtypes (WHO II–III) are strongly related to EBV infection, a known risk factor for NPC. Other predisposing genetic and environmental factors have resulted in a striking geographical distribution of NPC^2^. Treatment in the early stages of disease comprises radiotherapy and, more rarely, surgery, both of which can result in considerable morbidity^3^. Systemic treatment using platinum-based therapies is reserved for the recurrent and metastatic settings where the median survival ranges between 12 and 24 months^4^.

SSTR is a G protein-coupled cell surface receptor whose activation by extracellular ligands leads to inhibition of cell proliferation. In neuroendocrine tumors (NETs), SSTR2 expression is imaged with ^68^Ga-DOTA-peptide radioconjugates and therapeutically exploited by ^177^Lu- or ^90^Y-DOTA-peptide radioconjugates and SSTR2 agonists such as the octreotide octapeptide^5,6^. In NETs, SSTR2 receptor activation is associated with a decrease in proliferation, with SSTR2 agonists being cytostatic and effective at controlling growth but only in tumors with proliferation rates below 10%^7^. A previous study in five patients with primary EBV-positive NPC^8^ showed increased uptake of ^68^Ga-DOTA-TOC, with two case reports published around the same time, indicating the candidacy of SSTR expression for functional imaging of NPC^9,10^. In a study of 12 NPCs, SSTR autoradiography on tissue samples confirms these unexpected imaging results^11^. The ability of SSTR2 agonists to control NPC tumor growth is unknown. The sporadic reports suggesting a relationship between SSTR2 and NPC, its potential as a biomarker and the lack of a molecular explanation for SSTR2 expression in NPC prompted our larger study into the role of this receptor.

Here, we show that SSTR2 expression is a diagnostic and prognostic biomarker in NPC and is induced by the Epstein-Barr virus (EBV) latent membrane protein 1 (LMP1) via the NF-κB pathway. Furthermore, we demonstrate a key role of SSTR2 in imaging and targeted therapies.

### SSTR2 expression is EBV-linked in the majority of NPC

In order to validate the findings of previous studies we performed immunohistochemical staining of SSTR2 on 402 NPC primary, recurrent and metastatic tumor samples (detailed in Methods, Supplementary Table 1) from European and Asian centers as well as one US center. 252 of the 311 (81%; Supplementary Table 2) primary tumor samples showed SSTR2 expression, which was localized at the plasma membrane (a representative primary case is shown in Fig. 1a).

Where data on EBV status were available (*n* = 385 of 402 samples in total), 317/385 (82.3%) (Supplementary Table 3) of the patient cohort were found to be EBV-positive and to express EBV-encoded small RNAs (EBERs) (a representative example is shown in Fig. 1a). Interestingly, SSTR2 expression was significantly enriched in EBV-positive NPC (OR = 12.7; *p* < 0.001; Fig. 1b) and in the non-keratinizing histological subtypes (OR = 27.0; *p* < 0.001) and significantly associated with other clinicopathological factors (Fig. 1c and Suppl. Table 4). Furthermore, SSTR2 expression was maintained in local recurrent and metastatic disease (*n* = 91 of 402 samples in total), with no significant difference of expression levels between cases (Fig. 1c–e).

### EBV induces SSTR2 expression via LMP1 and *NF-ĸB* signalling

**Fig. 2:**
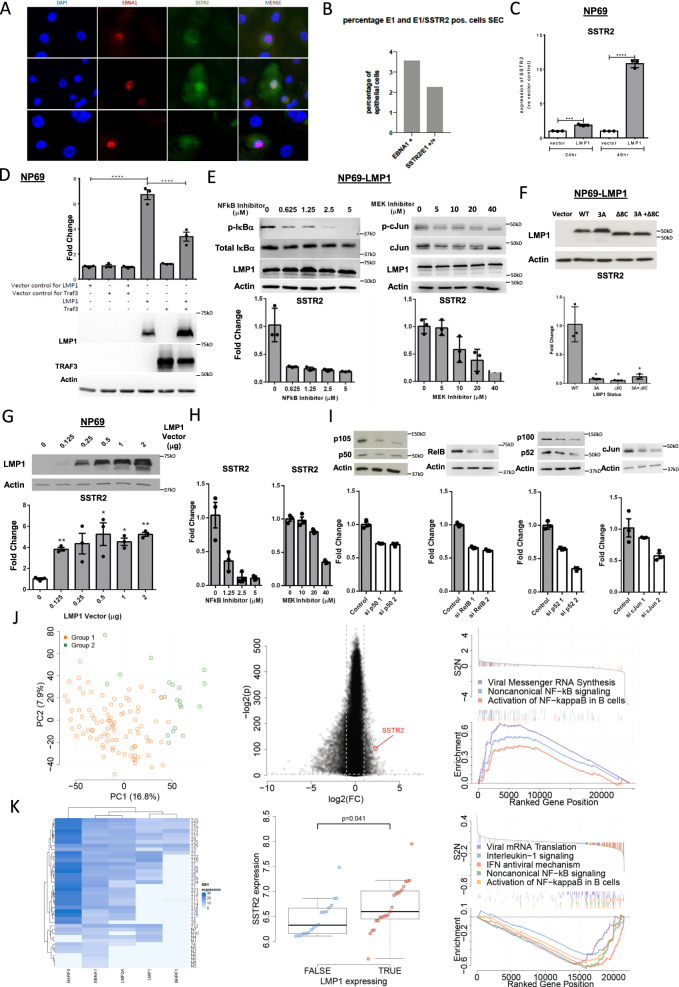
EBV infection is associated with SSTR2 upregulation in NPC—in vitro experiments and external validation dataset. **a** Immunofluorescence analysis of EBNA1 (red) and SSTR2 (green) expression in two representative examples of cultured primary respiratory epithelial cells before and after infection with the epitheliotropic M81 EBV strain. Nuclei are counterstained with DAPI (blue); Replication (*n* = 5). **b** Percentages of cells positive for EBNA1 or co-expression of SSTR2 and EBNA1 **c** LMP1 induces SSTR2 expression in NP69, an immortalized normal nasopharyngeal epithelial cell line (relative quantities of SSTR2 were calculated using the comparative threshold cycle method and normalized using human beta-actin as endogenous control. Data is presented as a ratio relative to vector control). **d** LMP1-mediated SSTR2 induction in NP69 cells is inhibited by ectopic expression of TRAF3, a negative regulator of NF-κB. **e** In NP69 cells, LMP1-induced SSTR2 expression is suppressed by the NF-κB inhibitor BAY 11-7085 and MEK inhibitor U0126. **f** Both CTAR1 and CTAR2 regions of LMP1 (Supplementary Fig. 2) are essential for LMP1-mediated SSTR2 induction. LMP1 mutant constructs 3 A, Delta 8 C and 3 A + Delta 8 C target CTAR1, CTAR2, and both regions respectively. **g** SSTR2 expression by LMP1 is dose dependent. **h** In C666-1 cells, LMP1-induced SSTR2 expression is suppressed by the NF-κB inhibitor BAY 11-7085 and MEK inhibitor U0126. **i** siRNAs mediated knockdown of the subunits of activated NF-κB signal complexes, NFκB1 (p105/p50), RELB, NFκB2 (p100/p52), or c-Jun in C666-1, both resulted in significant SSTR2 suppression. **j** Left panel, PCA on independent RNA-seq data of NPC (*n* = 113) identifies two groups of NPC tumors. The color of the samples is based on unsupervised hierarchical clustering. Middle panel, Differential gene expression analysis between Group 1 and 2 tumors shows that SSTR2 is highly-expressed in Group 1 tumors (log_2_ fold change = 2.3, adjusted *p* = 2.7e-32). Right panel, Pathway analysis demonstrates that Group 1 tumor show significant enrichment of viral biogenesis pathways. **k** Left panel, Heatmap of gene expression of EBV genes in an independent cohort of NPC (tumor *n* = 31, normal *n* = 10). Middle panel, Microarray SSTR2 expression is positively correlated with viral LMP1 expression (W = 129, *p* = 0.041, Wilcoxon two-sided test). Center line displays the median, boxes display the interquartile range. Whiskers display 1.5× the interquartile range. Outliers lie beyond the whiskers. Right panel, Pathway analysis demonstrates that LMP1-expressing tumor samples are enriched in viral biogenesis pathways. *, **, ***, and **** denote a significant difference between groups of *P* < 0.05, *P* < 0.01, *P* < 0.0001, and *P* < 0.0001, respectively. Source Data are provided as a Source data file.

We next explored a possible relationship of EBV infection and SSTR2 expression. EBV infection of cultured primary cells from normal respiratory epithelium led to a significant upregulation of SSTR2 expression (Fig. 2a, b and Supplementary Fig. 1). Aberrant activation of NF-κB signaling, either through expression of the LMP1 (latent membrane protein 1) oncoprotein of EBV or somatic mutation of negative regulators of NF-κB (e.g., in TRAF3, CYLD, NFKBIA, NLRC5) has been shown to play a driver role in NPC tumorigenesis^12–14^. Transient expression of LMP1 into the immortalized nasopharyngeal epithelial cell line NP69 induced *SSTR2* transcription (Fig. 2c, Supplementary Fig. 2a) indicating that EBV potentially upregulates SSTR2 expression through NF-κB signaling. This SSTR2 expression was suppressed by co-expression of TRAF3, a negative regulator of NF-κB (Fig. 2d) or by treatment with the NF-κB inhibitor BAY 11-7085 (Fig. 2e). Moreover, LMP1 proteins with mutant CTAR1 and CTAR2 domains, known to be critical for activation of NF-κB by LMP1 (ref. ^15^; Supplementary Fig. 2), induced significant lower SSTR2 expression compared with NPC transfected with wild-type LMP1 (Fig. 2f, g). In addition to contributing to NF-κB signaling, the CTAR1 domain of LMP1 also leads to activation of the AKT and MEK/ERK pathways. Using pharmacological inhibitors of these pathways indicated that LMP1-activated SSTR2 expression induced signaling by MEK but not AKT (Fig. 2e, Supplementary Fig. 2b). As a complementary approach to LMP1 transfection in NP69 cells, we used the C666-1 NPC cell line in which NF-κB signaling is known to be endogenously-activated via somatic mutation of its negative regulators TRAF3, CYLD, and TNFAIP3^12,13^. SSTR2 expression in C666-1 cells was suppressed by the NF-κB inhibitor BAY 11-7085 and the MEK inhibitor U0126 (Fig. 2h) or upon siRNA-driven downregulation of subunits of activated NF-ĸB signal complexes (NFKB1 (p105/p50), NFKB2 (p100/p52) or RELB) and c-Jun (Fig. 2i, Supplementary Fig. 2c, d)^13^. Taken together, these findings imply that EBV infection induces SSTR2 expression in nasopharyngeal epithelial cells through expression of the latent oncoprotein LMP1 and activation of the NF-κB and MEK signaling pathways (Supplementary Fig. 3). This observation was confirmed further in other EBV-induced cancers (Supplementary Fig. 4).

### EBV-driven signalling pathways involved in SSTR2 expression

To validate our finding of an association between SSTR2 expression and EBV infection, we analyzed an independent NPC cohort where gene expression data were available for 113 samples^16^. Unsupervised hierarchical clustering and principal component analysis (PCA) based on gene expression revealed two groups of samples in this dataset (Fig. 2j, left panel). Differential gene expression analysis between these groups revealed overexpression of SSTR2 in group 1 tumors (Fig. 2j, middle panel). Furthermore, geneset enrichment analysis (GSEA) revealed an upregulation of pathways related to viral infection in group 1 samples compared to the other group (Fig. 2j, right panel). Further, a supervised analysis on another dataset where both EBV gene expression (Fig. 2k, left panel) and microarray human gene expression^17^ were available, revealed a positive correlation between viral LMP1 expression and tumor SSTR2 expression (Fig. 2k, middle panel), tumor NFκB1 expression and SSTR2 expression (Supplementary Fig. 5) and upregulation of viral biogenesis pathways in LMP1-expressing samples compared to non-LMP1-expressing samples (Fig. 2k, right panel).

### Antitumour effect of PEN-221, an anti-SSTR2 drug conjugate

**Fig. 3:**
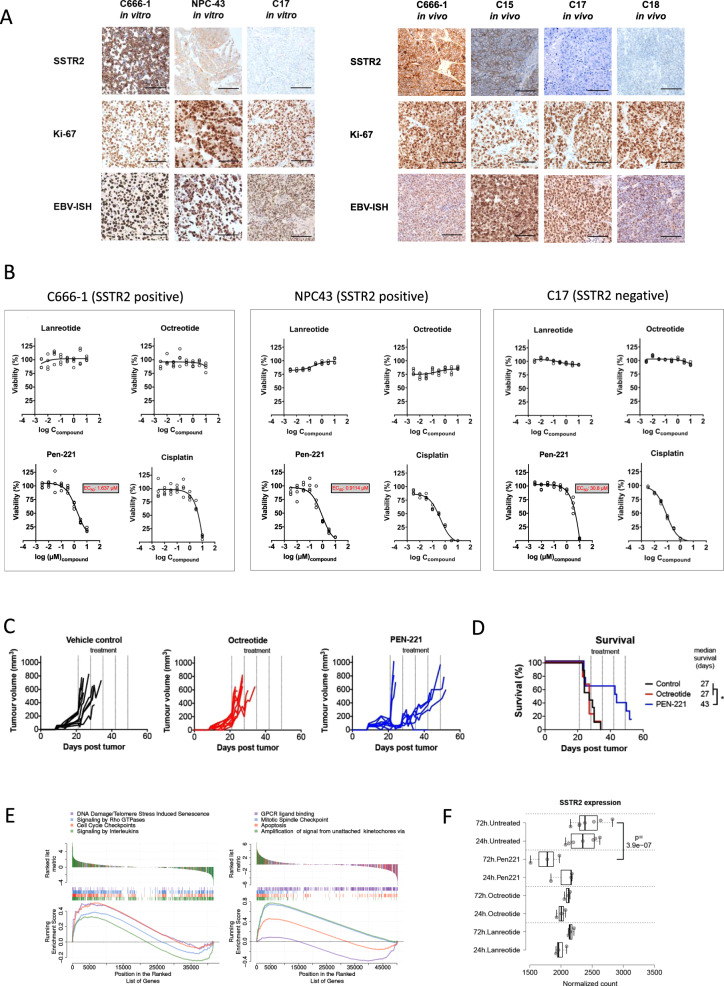
In vitro and in vivo effects of SSTR2 agonists on the C666-1 NPC cell line. **a** Immunohistochemical characterization of C666-1, NPC43, and C17 cell lines cultured in vitro and from xenografted C666-1, C15, C17, or C18 tumor tissues (scale bar 100 µm); Replication *n* = 2. **b** In vitro dose response curves and half-maximal effective concentration (EC50 values) of the indicated SSTR agonists on C666-1, NPC43, and C17 cells. EC50 = half-maximal effective concentration. **c** Growth curves of C666-1 tumors in nude mice treated with vehicle (*n* = 9), octreotide (*n* = 9), or PEN-221 (*n* = 8). The dotted lines indicate the time points of drug injection. **d** Kaplan–Meier curves of athymic nude mice with C666-1 tumors, treated with vehicle control (*n* = 9), octreotide (*n* = 9), or PEN-221 (*n* = 8), with dotted lines showing time points of drug or vehicle injection (*p = 0.0368; two-sided Log-rank Mantel-Cox test). **e** Geneset enrichment analysis reveals upregulation of senescence pathways 24 h post lanreotide treatment (left), and upregulation of apoptosis and mitotic spindle assembly pathways 24 h post-PEN-221 treatment (right) in treated vs untreated cell lines. **f** mRNA sequencing analysis of C666-1 cells treated in vitro (72 h with PEN-221) reveals downregulation of SSTR2 expression (two-sided Wald test, adjusted *p* = 3.9e-7). Center line displays the median, boxes display the interquartile range. Whiskers display 1.5× the interquartile range. Source Data are provided as a Source data file.

We then hypothesized that SSTR2 expression observed in patients may sensitize NPC to SSTR2-targeted cytostatic or cytotoxic agents. To investigate this, we used the well-characterized EBV-positive NPC cell lines C666-1^18^, NPC43^19^, and C17^20^. When taken from in vitro cultures or in vivo xenografts, C666-1 and NPC43 showed high expression of EBERs, SSTR2 and the Ki-67 proliferation antigen whereas C17 was found to be SSTR2 negative (Fig. 3a). We next tested the impact on in vitro proliferation/survival of these cell lines of cisplatin, a chemotherapeutic agent used in the treatment of patients with NPC, and a range of SSTR2 agonists, including the FDA-approved lanreotide and octreotide and PEN-221, which is in Phase 1/2a clinical trial in the UK and US for NET patients^21^. PEN-221 is a drug conjugate made up of a peptide that is highly selective for SSTR2 conjugated to the tubulin polymerization inhibitor DM1 and has shown effectiveness in preclinical models^22^. Unlike in NETs, lanreotide and octreotide did not affect in vitro proliferation of C666-1 and NPC43, in contrast to PEN-221 (Fig. 3b and Supplementary Fig. 3). We next subcutaneously xenografted C666-1 cells into athymic nude mice and treated established tumors with octreotide (*n* = 9), PEN-221 (*n* = 8), or vehicle control (*n* = 9). Mice treated with PEN-221 showed a significant increase in overall survival (*p* = 0.0368; Log-rank Mantel-Cox test) (Fig. 3c, d), further indicating superior anti-tumor efficacy of PEN-221 as compared to octreotide.

### Molecular effects of in vitro cytotoxic payload of PEN-221

We next performed RNA-seq to explore the molecular effects of SSTR2-targeted drugs on C666-1 cells. Lanreotide and octreotide did not affect SSTR2 expression, but induced upregulation of pathways related to somatostatin biology such as interleukin signaling^23^ as well as upregulation of cell senescence pathways 24 h post-treatment (Fig. 3e shows data for lanreotide, data for octreotide not shown), but no changes in cell death/apoptotic pathways. In contrast, 72-h treatment with PEN-221 led to significant downregulation of SSTR2 expression (Fig. 3f) as well as upregulation of pathways related to apoptotic signaling and mitotic spindle formation dysregulation, the latter in keeping with the mechanism of the cytotoxic payload of PEN-221^24^.

### SSTR2 as a diagnostic NPC biomarker in the clinic

**Fig. 4:**
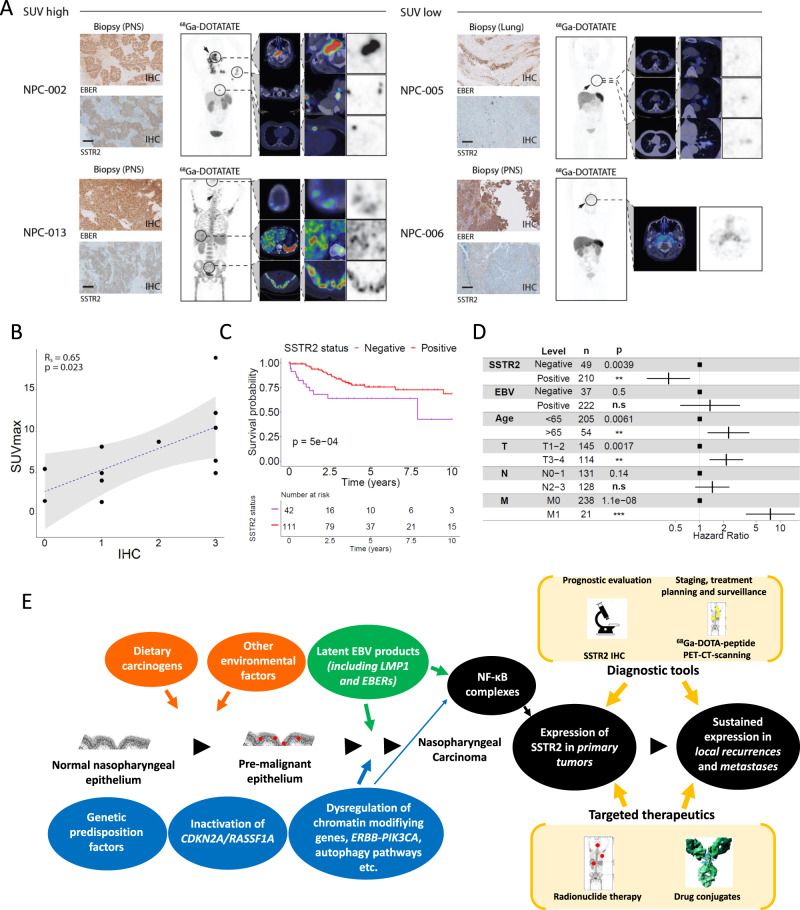
Visualization and prognostic value of SSTR2 expression in NPC patients. **a** Visualization of SSTR2 expression by ^68^Ga-DOTA-TATE PET-CT imaging (clinical characteristics and SSTR2 status of NPC patients undergoing ^68^Ga-DOTA-TATE PET-CT imaging are shown in Supplementary Table 4). **b** Correlation of SSTR2 expression with in vivo uptake of ^68^Ga-DOTA-TATE. SSTR2 IHC score shows significant positive correlation to SUVmax of biopsied lesion. (black circles: biopsied lesions, *n* = 12; Spearman’s correlation coefficient: R_s_ = 0.65; *p* = 0.023). **d** SSTR2 expression status remains prognostic independent of EBV status, age, and primary tumor (T), lymph node (N), and metastasis (M) staging. n.s not significant; *p < 0.05; **p < 0.01; ***p < 0.001. Vertical lines display the hazard ratio estimate, horizontal lines display the 95% confidence interval. **e** Proposed model of NPC oncogenesis and cancer progression involving EBV and SSTR2 expression. In the multistep carcinogenesis of NPC, inactivation of tumor suppressor genes is believed to occur prior to EBV infection and to be induced by dietary carcinogens and other environmental factors. Infection of nasopharyngeal cells with EBV and establishment of a latent infection probably occurs at a late stage in the acquisition of the malignant phenotype. Genetic alterations identified in premalignant nasopharyngeal epithelium may play crucial roles to support stable EBV infection. Once a premalignant cell has been infected by EBV, it appears to rapidly evolve towards an invasive tumor, with the stage of EBV-positive in situ carcinoma being very transient. SSTR2 expression is acquired following the onset of latent EBV infection by LMP1 expression via NF-ĸB signaling. On the basis of this tentative scenario, pharmacological agonists of SSTR2 are expected to provide the maximal benefit for three types of indications: (1) as part of the initial curative treatment of the primary tumors, (2) as part of adjuvant treatment following clinical remission of the primary tumor; (3) with a prophylactic intent for subjects at risk of NPC manifested by EBV serological changes and/or increasing circulating EBV DNA load. Source Data are provided as a Source data file.

In a clinical trial of NPC patients (NCT03670342) we show the use of SSTR2 as a potential noninvasive biomarker in NPC and integrated SSTR2 protein expression data with ^68^Ga-DOTA-peptide imaging data on 12 patients. We found a significant correlation of SSTR2 expression levels with in vivo uptake of ^68^Ga-DOTA-peptides (Fig. 4a, b), indicating the potential of this imaging modality as a noninvasive marker to monitor SSTR2 expression and as a target for SSTR2 receptor-targeted radionuclide therapy (Lutetium-177, Ytrium-90).

### SSTR2 expression is a prognostic biomarker in NPC

Analysis of overall survival of the study population in patients from the European centers (where standards of treatment were comparable) revealed improved survival in NPC patients with tumors positive for SSTR2 (*p* < 0.001) (Fig. 4c). Jointly classifying patients by EBV and SSTR2 status revealed the poorest prognosis for those patients who are both EBV negative and SSTR2 negative (Supplementary Fig. 7). Further, in a multivariate cox regression analysis with SSTR2 status, EBV status, patient age, T, N, and M staging in all patients for which such information was available (*n* = 209), SSTR2 positivity remained predictive of an improved prognosis for patients (HR = 0.41) (Fig. 4d). Importantly, in this analysis, EBV status was not prognostic independently of SSTR2 status.

## Discussion

In summary, this comprehensive analysis of SSTR2 expression in 402 primary, local recurrent and metastatic NPC patient samples established a highly significant association with EBV infection and suggest a key role for this receptor in this tumor (positive in >80% of samples). By integrating the mechanisms of SSTR2 expression, preclinical studies and imaging findings, we propose an updated model of NPC carcinogenesis in parallel with potential diagnostic, prognostic, monitoring, and therapeutic strategies (Fig. 4e; related literature can be found in Refs. ^25–28^).

We also demonstrate the potential of SSTR2 expression for prognosis and application with ^68^Ga-DOTA-peptide scanning, akin to the application of this imaging technique in gastrointestinal NETs and prostate, breast, and colorectal cancers^7,29,30^. The significant correlation of SSTR2 expression levels with in vivo uptake of ^68^Ga-DOTA-peptide indicates that this imaging modality may be used as a noninvasive marker to select patients for PEN-221 treatment, monitor its response and possibly surveillance of NPC, surgical and radionuclide therapy planning and as an application for intraoperative navigation systems via integration of functional imaging data.

Following recent data from an open label phase III multinational trial^31^, gemcitabine plus cisplatin is currently the standard of care in recurrent or metastatic NPC. Having identified SSTR2 expression in the majority of NPCs and preclinical efficacy of SSTR-targeted therapies, our data support inclusion of NPC in the ongoing Phase 1/2a clinical trial of PEN-221 in NETs.

In conclusion, our study has established SSTR2 as an EBV-induced druggable target in primary, recurrent, and metastatic NPCs and the preclinical efficacy of a targeted therapy. We demonstrate the mechanistic link of EBV infection and SSTR2 expression and provide a rationale for routine testing of SSTR2 status in NPC patients for prognostic purposes, for exploration of the value of 68Ga-DOTA-peptide PET-CT imaging for NPC diagnosis, surveillance and surgical planning and for offering a theragnostic treatment for advanced and recurrent NPC.

## Methods

### Materials

Four hundred and two formalin-fixed paraffin-embedded (FFPE) NPC specimens were obtained from three European institutions (University College London/University College London Hospital, UK; Medical University of Innsbruck, Austria; University Medical Center in Utrecht/UMCU, Netherlands), one US institution (Stanford University, Palo Alto, US) and from four institutions in Asia (Gadjah Mada University/Dr. Sardjito Hospital, Yogyakarta, Indonesia, The Chinese University of Hong Kong, Hong Kong SAR, Jinan University, Shenzhen, Guangdong, China, and National Cancer Centre Singapore). Ethical approval was obtained from all institutions (Yogyakarta, Indonesia: KE/FK/0198/EC/2017; Singapore: 2015/2482; Hong Kong, China: CREC-2013-229; Shenzhen, China: LL-KY-2019143; London, UK: UCL 04/0099; REC 04/Q0505/59; Stanford, US: IRB-43567; Innsbruck, Austria: AN2014-0241, 340/4.20; Utrecht, Netherlands: TCBio 14/510) with further ethical approval for multicenter data analysis from University College London Research Ethics Committee (UCL REC no. 9609/002). Histological characterization and sample selection/cohort was performed by two head and neck pathologists (SWI; SS) both experienced in the evaluation of NPCs. The date of diagnosis was defined as the date of tissue extraction for histological determination of the diagnosis. The end date for OS was the date of death.

### Tissue microarray construction

Tissue microarrays (TMA) were constructed from the 93 specimens from University Medical Center in Utrecht/UMCU, Netherlands. The TMAs were constructed with a TMA Grand Master instrument (3D HISTECH, Budapest, Hungary) using the respective FFPE blocks. Tumor areas were marked by a pathologist (SWI) and pathology resident (MOO) experienced in the histological evaluation of NPCs. Three cores (0.6 mm) were punched from the marked tumor areas and arrayed into a recipient TMA donor block^32^.

### Immunohistochemical analysis of expression of SSTR2, Ki-67 Chromogranin A and Synaptophysin

Immunohistochemistry was performed in different institutions, almost all using Ventana automated staining instruments (Ventana Medical systems, Tuscon, AZ, USA). The Singapore team used a Leica Bond-Max (Leica Biosystems, Wetzlar, Germanny) autostainer for these purposes. For detection of chromogranin A (LK2H10; Ventana), synaptophysin (SP11; Ventana) and Ki-67 (Clone MIB-1, DAKO, Glostrup, Denmark), routinely available staining protocols were used. For detection of SSTR2, the rabbit monoclonal antibody UMB1 (Abcam, Cambridge, UK) was used. More detailed staining protocols are listed in the Supplementary Material. In Utrecht an anti-SSTR2 antibody (rabbit polyclonal, code SS-8000-RM, diluted 1:5, BioTrend, Cologne, Germany) was used during the initial run and further validated with the above antibody (Supplementary Fig. 7). The slides were evaluated under the guidance of head and neck pathologists (SWI, SS). The evaluators of the immunohistochemical stains were blinded to the clinical outcomes or EBV status and membranous staining of the tumor cells was assessed (Fig. 1). The slides were dichotomously scored as being positive or negative, based on the extent of staining and intensity. The extent was scored on a continuous scale from 0–100%. The intensity was scored as three categories (1: weak staining not easily seen via the low power objective; 2: moderate staining still seen on a low power objective; 3: strong staining easily visible via a low power objective).

### EBV status

EBV status was determined by Epstein-Barr virus-encoded Early RNA (EBER) in situ hybridization (ISH) on the samples, brushes or TMA. Ventana BenchMark automated staining instruments (Ventana Medical systems, Tuscon, AZ, USA) were used for ISH of the samples or TMA using an EBV-specific probe (INFORM EBER PROBE; Ventana Medical systems) and ISH iVIEW Blue detection kit (Ventana Medical systems, Inc.) for staining using the manufacturer’s instructions in Innsbruck, Utrecht, Hong Kong, Stanford and London. Shenzhen used an EBER Probe (Zhongshan Jinquaiao Biotechnology Co.; Beijing, China) and an autostainer (Ventana Medical Systems, Inc.) to perform ISH. Singapore used a BOND^TM^ Ready-to-use ISH EBER probe and a Leica Bond-Max autostainer (all Leica Biosystems, Wetzlar, Germany) for this purpose. In situ hybridization of xenografts and cell pellets was done using an EBV-specific probe (INFORM EBER PROBE; Ventana Medical systems) and ISH iVIEW Blue detection kit (Ventana Medical systems, Inc.) using the manufacturer’s instructions.

### EBV infection of primary epithelial cells

Primary epithelial cells were grown on glass slides in serum-free keratinocyte growth medium (KGM-SFM, Thermo Fisher Scientific, US) at 37 °C, 5% CO_2_. Cells were exposed to 5 × 10^7^ M81 viruses for 72 h, followed by virus removal and washing of the cells with 1× PBS. The slides were dried, fixed with 4% PFA for 15 min, permeabilized for 10 min in 1× PBS/0.1%Triton-X100. After a 30 min blocking step and cells being kept in in 1% BSA/PBS at 37 °C for 4 min, the slides were incubated with a rabbit antibody directed against human SSTR2 (clone 11HCLC, Thermo Fisher; dilution 1:200 in 1% BSA/PBS) for 12 h at 4 °C. The slides were then washed and incubated with a rat antibody specific for EBNA1 (clone 1H4, provided by R. Feederle, Munich, Germany; dilution 1:10 in 1% BSA/PBS) for 2 h at 37 °C in a humidified chamber. After three washes steps, the cells were incubated with a secondary goat anti-rabbit antibody coupled to Alexa 488 (A11008, Invitrogen; dilution 1:300) and with a secondary goat anti-rat antibody coupled to Cy3 (112-165-143, Dianova; dilution 1:900) for 30 min at 37 °C. Cell nuclei were counterstained with DAPI (40 ng/ml). Slides were analyzed using a Leica epifluorescence microscope equipped with a CCD camera. qPCR: After DNAse treatment, Trizol-purified RNA was reverse transcribed with AMV-reverse transcriptase (Roche) using a mix of random primers. EBER transcripts were detected by quantitative PCR using specific primers (EBER1 fwd 5’-acgctgccctagaggttttg-3’, EBER1 rev 5’-gcagaaagcagagtctggga-3’) and probes (EBER1 probe 5’FAM-aggacggtgtctgtggttgt-3’TAMRA) for 40 cycles using the universal thermal cycling protocol on an ABI STEP ONE PLUS Sequence Detection System (Applied Biosystems). All RT–PCRs included samples not treated with reverse transcriptase that served as negative controls. All samples were run in duplicate, together with primers specific for the human *GAPDH* gene to normalize for variations in cDNA recovery. SSTR2 transcripts were amplified using specific primers (SSTR2 fwd GAAGAGAATCAATAGCGTGTTTTATTGCATGTC, SSTR2 rev CATAGCGGAGGATGACATAAATGAC) for 40 cycles. Non-infected primary epithelial cells served as a negative control. All used primers are listed in supplementary Table 6.

### Culture of the cell lines

C666-1 cells were cultured in RPMI-1640 with 25 mM Hepes (Lonza, Berlin, Germany) supplemented with 10% heat-inactivated FCS (Gibco, Carlsbad, CA), 2 mM L-Glutamine, 100 units/ml penicillin (Gibco), and 0.1 mg/ml streptomycin (Gibco). Cells were maintained at 37 °C, 5% CO_2_ and passaged every 7 days at a 1:2 ratio using accutase (Sigma, St. Louis, MO, USA). Seventy-five percent of culture medium was replaced by fresh medium every 2–3 days. NPC43 and C17 cell lines were cultured in RPMI-1640 with 25 mM Hepes (Lonza, Berlin, Germany) supplemented with 10% heat-inactivated FCS (Gibco, Carlsbad, CA), 2 mM L-Glutamine, 100 units/ml penicillin (Gibco) and 0.1 mg/ml streptomycin (Gibco) and 4 µM Y27632 (Promocell, Heidelberg, Germany). Cells were maintained at 37 °C, 5% CO_2_ and passaged 5 days at a 1:4 ratio using accutase (Sigma, St. Louis, MO, USA). Culture medium was replaced by fresh medium every 2 to 3 days.

### Immunohistochemical analysis of SSTR2 in cell pellets

Routinely cultured cell lines (2–4 × 106) were collected by centrifugation and embedded as cell pellet in agarose as published before (ref. ^33^, modified as follows^34^): Cells were harvested by centrifugation at 290 × *g* for 10 min at 4 °C, and the resulting pellet was fixed in 10 ml neutral-buffered 4% formaldehyde solution (Flintsbach am Inn, Germany). After fixation the cells were centrifuged by 400 × *g* for 10 min at room temperature. The cell pellet was resuspended in 300 µl PBS, transferred to Eppendorf tube (1.5 ml), and kept on ice. Low melting point agarose (with gelling temperature point 34–37 °C) was prepared in PBS as 3% solution in labor glassware by microwave warming and equilibrated in a thermoblock to 65 °C for at least 30 min. The 300 µl PBS—cell suspension was also equilibrated to 65 °C for not more than 10 min. 600 µl melted equilibrated agarose was pipetted to the cell suspension, followed by spinning at 2000 × *g* for 5 min at room temperature. After that, the tube was placed on ice, the cell pellet was trimmed and was placed in embedding cassette. The cell pellet in the cassette was stored in PBS containing 0.05–0.1% sodium azide until embedded in paraffin in a Histos 5 (Histocom, Wr. Neudorf, Austria) paraffin embedding system, following the instructions of the manufacturer. After embedding, biopsies were sectioned and used for in situ hybridization and immunohistochemistry. Embedded specimens were serially sectioned at 5 µm thickness using a HM 355 S microtome (Microm, Walldorf, Germany) and affixed onto SuperfrostTM Plus slides (Menzel, Braunschweig, Germany). The mounted specimens were then dried overnight at room temperature, following which the slides were incubated at 60 °C for 1 h to enable the sectioned specimens to adhere firmly onto the glass surface. Immunohistochemical analysis of SSTR2 expression was performed as above.

### Drugs

Lanreotide and octreotide acetate were obtained from Abcam® (Cambridge, UK). Belinostat (PXD101) was obtained from Selleckchem (Houston, US), Cisplatin ‘Ebewe’ from Sandoz (Holzkirchen, Germany) and PEN-221^35^ was provided by Tarveda Therapeutics (Watertown, Massachusetts, US).

### MTT-based cell viability assay

Two days prior addition of test compounds, NPC cells were plated at a density of 2 × 10^4^ cells/well in 96-well plates. Compounds were prepared as advised by manufacturers, diluted half logarithmic in medium and added to the wells in quadruplicate starting from 10 µM (30 µM for cisplatin). PEN-211 containing medium was removed after 2 h, cells were washed with PBS and fresh medium was added. Plates were incubated in a humified chamber for 7 days; 50% of medium were exchanged every other day with fresh drug dilutions. MTT (3-[4,5-dimethylthiazol-2-yl]−2,5 diphenyl tetrazolium bromide) reagent (20 µl/well of 5 mg/ml solution) (Sigma–Aldrich) was added and solubilized after 4 h using 100 µl of a 0.1 mg/ml SDS/0.01 M HCl solution. Dual absorbance was measured after 5 h in a microplate reader (Epoch BioTek, BioTek Instruments, Bad Friedrichshall, Germany) using 550 nm as measurement and 655 nm as reference filter. After subtraction of background absorbance, fractions of surviving cells were obtained by normalization to the mean of nontreated samples.

### RNA-seq analysis

C666-1 cells were seeded at a density of 1 × 10^6^ /well in six-well plates 2 days prior to compound addition. PEN-221 was prepared as advised by the manufacturer and 5 µM were added in triplicates. Untreated and DMSO-treated (1:2000 in medium) wells were used as controls. 24 and 72 h post-PEN-221 addition, DNA and RNA extraction was performed from separate wells using Quick-DNA Plus and Direct-zol RNA Plus Kit (Zymo Research, Irvine, CA, USA) according to the manufacturers’ instructions.

RNA concentration was normalized to 100 ng/50 µl and Illumina libraries prepared using a NEBNext Poly(A) mRNA Magnetic Isolation Module in conjunction with a NEBNext Ultra II Directional RNA Library Prep Kit (New England Biolabs), according to the manufacturer’s instructions using adapters diluted 1:50. Libraries were quantitated using the Agilent High Sensitivity D1000 ScreenTape System on a 2200 Tapestation (Agilent), pooled at equimolar concentration, denatured and sequenced on a NextSeq 500 (Illumina).

RNA sequencing transcript abundance was estimated from fastq files using the reference-free quantification tool salmon^36^ with the gencode GRCh37 transcript annotation. Differential expression analysis was performed using DESeq2^37^ with treatment, time and batch (where applicable) as covariates. Geneset enrichment analysis was performed using the GSEA function from clusterProfiler^38^, with the reactome database^39^ pathways on the gene DESeq2 Wald statistics and a minimum and maximum pathway size of 25 and 500, respectively. Pathways with a q-value <0.05 were considered significantly enriched.

For publicly-available NPC dataset where only FPKM values were available, differential expression analysis was performed using the limma package^40^ with sample groups defined through hierarchical clustering of the log2(FPKM + 1) values. Adjusted p-values were computed using the eBayes function. GSEA was performed as for DESeq2 results, but with log2-fold change as the ranking score.

### In vivo mouse experiments

The experiments have been approved by the Animal Welfare and Ethical Review Body (AWERB) of University College London according to Animals (Scientific Procedures) Act 1986 under the project license of B.V. and personal licenses of scientists involved at UCL Optimisation, growth curves and SSTR2 staining were performed in preparation of the planned experiments. 2 × 10^6^ C666-1 cells were subcutaneously injected in the right flank in 100 µl (50% Matrigel) of 6–8 week-old female athymic nude mice with a starting weight of around ~20 g. Mice are kept within Home Office limits of 18–22 °C and 40–60% humidity. The mice run on a 12 h light/dark cycle that from 7 am to 7 pm. Three groups of 10 mice each were used. PEN-221 was administered at a dose level as per previous MTD study (detailed below) with PEN-221 vehicle buffer and octreotide as negative controls. Drug treatment was initiated when median tumor volume was >0.1 cm^3^ and mice were monitored twice weekly. Mice were scored for health, weighed and tumors were measured via calipers. Mice were culled when the humane endpoint was reached when either tumors reached >1 cm^3^, there were tumor ulcerations, or weight loss >20%.

Octreotide was formulated in 0.5% solutol/5% Mannitol/5 mM Acetate buffer, pH 4.0 at a concentration of equimolar to the concentration of PEN-221. Vehicle control (PEN-221 vehicle buffer) was 0.5% Solutol/5% Mannitol/5 mM Acetate buffer, pH 4.0. PEN-221: PEN-221 was formulated in 0.5% Solutol/5% Mannitol/5 mM Acetate buffer, pH 4.0 at a concentration to support dosing at 1.5 mg/kg, i.e., 0.30 mg/ml for 5 ml/kg.

Treatment was started when median tumor volume ≥0.1 cm^3^. Mice were allocated into three groups (1) Control: 5 × weekly injection at a dosing volume of 5 ml/kg; (2) Octreotide: 5 × weekly injection at a dosing volume of 5 ml/kg of the solution prepared at a concentration that is the molar equivalent to the dose of PEN-221; (3) PEN-221: 5 × weekly injection at a dosing volume of 5 ml/kg of the solution prepared at an agreed upon selected dose identified from Study A above. C666-1 were xenografted and tumor specimens were stained on formalin-fixed paraffin-embedded slides as described above for SSTR2.

### Patient survival analysis

IBM SPSS Statistics software (version 24) and R was used to analyze the data. The likelihood of univariable independence between groups was performed using the Pearson X^2^ test (and the Fisher’s exact test when appropriate) for categorical variables. Rates of survival were calculated with the Kaplan–Meier method and comparison of survival by Log-rank test. SSTR2 positivity was compared using backward logistic regression analysis, also taking into account significant clinicopathological characteristics. The following clinicopathological characteristics were dichotomized: age (cutoff at 65 years), T-stage (T1/2 versus T3/4), N-stage (N0 *versus* N1/2/3), and M-stage (M0 versus M1). Two-tailed *p*-values <0.05 were considered statistically-significant.

Multivariate cox regression survival analysis was performed with the R package survival, with center (dichotomized to Europe/Asia), SSTR2 status, EBV status, sex, and age as well as T (dichotomized to T1-2 and T3-4), N (dichotomized to N0-1 and N2-3), and M staging as covariates initially. Center and sex were removed from the final model as they were found to be nonsignificant (*p* < 0.05). The proportional hazards assumption was tested using the cox.zph function; none of the covariates in the final model were found to violate the proportional hazards assumption (*p* < 0.05). Concordance was calculated with Cohen’s Kappa.

### ^68^Ga-DOTATATE molecular Imaging

Patients were recruited prospectively under the Pilot study of Somatostatin Receptor Imaging in Nasopharyngeal carcinoma (ClinicalTrials.gov: NCT03670342). Informed consent was obtained from all patients, and approval was obtained from the centralized institution review board (IRB protocol no. 2015/2482).

Peptide labeling with ^68^Ga in the Department of Nuclear Medicine and Molecular Imaging, Singapore General Hospital was performed using an automated synthesis module (Scintomics GmbH). In brief, 40 µg DOTA-[Tyr^3^] Octreotate precursor (Auspep) was radiolabeled with Gallium-68 eluted from ^68^Ge/^68^Ga generator (iThemba, South Africa). The 68Ge/68Ga generator is eluted with 10 ml 0.6 M hydrochloric acid. Gallium-68 was trapped using PS-H cartridge and eluted with 1.7 ml 5 M sodium chloride solution into 3 ml of 1.5 M HEPES buffer solution. The solution was heated at 125 °C for 6 min and purified using a Sep-Pak Light C18 cartridge and the final labelled product eluted with 2 ml of 50% Ethanol (v/v) into a product vial and diluted with 19 ml of phosphate buffered saline. Radiochemical purity was established by thin layer chromatography and the purity exceeded 90% in all cases.

Whole-body ^68^Ga-DOTATATE and ^18^F-FDG PET/CT imaging was performed using a dedicated General Electric PET/CT system (GE Discovery 690 VCT, GE Medical Systems, LLC, Waukesha, Wisconsin USA). Scans were acquired from the skull vertex to mid-thighs. Computed tomography (CT) was performed with (for 18F-FDG PET) and without (for 68Ga-DOTATATE PET) intravenous contrast media application for attenuation correction purposes as follows: 100 kV; “GE smart mA dose modulation” helical thickness: 3.75 mm; table speed: 0.984 mm; rotation time: 0.8 sec. Attenuation-corrected whole-body (vertex of skull to upper thighs) scans were acquired in 3-dimensional mode (2-min emission time per bed position). Depending on body length, eight bed positions were used with a field of view of 50 cm. For iterative reconstruction of the time of flight (TOF)-data, three iterations and 24 subsets with a filter cutoff of 7.0 were used. The interval between PET scans was not more than 7 days.

Patients received an intravenous injection of 111–148 MBq of ^68^Ga-DOTATATE and the acquisition was started after an uptake time of at least 60 min according to previously described imaging protocols. Multiplanar reconstruction was performed for image interpretation. Images were interpreted by an experienced nuclear medicine physician. A PET score was obtained, categorizing the uptake qualitatively as none, mild, moderate, and strong. In addition, semiquantitative measurements of intensity of tracer uptake were performed using the maximum standardized uptake value (SUVmax). For SUVmax calculations volumes of interest of tumor lesions (nasopharyngeal tumor, nodes, and metastatic sites including lymph nodes, soft tissue, visceral, and osseous lesions) and reference tissue (liver and spleen) were drawn automatically and adjusted manually to lesion size, applying a commercially available software provided by the vendor of the PET/CT-scanner (GE AWS).

### Reporting summary

Further information on research design is available in the Nature Research Reporting Summary linked to this article.

## Supplementary information

Supplementary Information

Reporting Summary

## Data Availability

The RNA sequencing data from untreated/treated NPC cell lines have been deposited in the GEO database under the accession code GSE160882. The expression array data referenced during the study are available in a public repository from the GEO website under accession GSE12452. The RNA sequencing data references during the study are available from the GEO website under accession GSE102349. All the other data supporting the findings of this study are available within the article and its supplementary information files and from the corresponding author upon reasonable request. Source data are provided with this paper.

## Source data

Source Data
