# Supplementary material for: Somatostatin receptor 2 expression in nasopharyngeal cancer is induced by Epstein Barr virus infection: impact on prognosis, imaging and therapy
Source: Nat Commun. 2021 Jan 5;12:117. doi: 10.1038/s41467-020-20308-8 (PMC7785735; doi:10.1038/s41467-020-20308-8)
Supplement: Supplementary file 1 — Supplementary Information [file 41467_2020_20308_MOESM1_ESM.pdf]

Supplementary Information

Somatostatin receptor 2 expression in nasopharyngeal cancer is induced by Epstein Barr virus infection: impact on prognosis, imaging and therapy

Supplementary Tables:

		N	%
Sex			
	Male	285	70.9
	Female	111	29.1
	Total	402	100.0
Age categorized			
	<65	325	80.8
	≥65	77	19.2
	Total	402	100.0
Type of histology			
	WHO type I	20	5.0
	WHO type II	75	19.0
	WHO type III	299	75.9
	Total	394	100.0
EBV status			
	Positive	317	82.3
	Negative	68	17.7
	Total	385	100.0
Tumor stage			
	T1	92	25.8
	T2	95	26.6
	T3	75	21.0
	T4	95	26.6
	Total	357	100.0
Nodal stage			
	N0	76	21.1
	N1	99	27.4
	N2	129	35.7
	N3	57	15.8
	Total	361	100.0
M stage			
	M0	296	91.6
	M1	27	8.4
	Total	323	100.0
UICC classification			
	Stage I	15	4.7
	Stage II	67	20.9
	Stage III	108	33.8
	Stage IVA	100	31.3
	Stage IVB	30	9.4
	Total	320	100.0

Supplementary Table 1: Characteristics of the NPC tissue samples investigated in this study. SSTR2 expression was found to be significantly-enriched in EBV-positive NPC (OR=12.7; p<0.001), in the non-keratinizing histological subtypes (OR=27.0; p<0.001), higher N stage (OR=2.3, p=0.003). No correlation was found with sex, T-stage, M-stage and overall UICC-stage.

	SSTR2 staining intensity							
	Primary		Local recurrence		Metastasis		Total	
	N	%	N	%	N	%	N	%
Strong	128	41.2	30	52.6	17	50.0	175	43.5
Moderate	80	25.7	8	14.0	9	26.5	97	24.1
Weak	44	14.1	6	10.5	4	11.8	54	13.4
Negative	59	19.0	13	22.8	4	11.8	76	18.9
Total	311	100.0	57	100.0	34	100.0	402	100.0

Supplementary Table 2: SSTR2 expression (assessed by semi-quantitative IHC scoring) of the NPC tissue samples investigated in this study.

	SSTR2 status (n)		Total	p-value
	Positive	Negative		
London				
EBV positive	11	4	15	0.52
EBV negative	1	1	2	
Missing	0	0	0	
Total	12	5	17	
Innsbruck				
EBV positive	30	0	30	$3.2e^{-7}$
EBV negative	5	11	16	
Missing	0	0	0	
Total	35	11	46	
Utrecht				
EBV positive	54	4	58	$4.9e^{-9}$
EBV negative	11	21	32	
Missing	2	1	3	
Total	67	26	93	
Yogyakarta				
EBV positive	34	4	38	0.45
EBV negative	8	0	8	
Missing	10	0	10	
Total	52	4	56	
Shenzhen				
EBV positive	26	2	28	0.19
EBV negative	1	1	2	
Missing	0	0	0	
Total	27	3	30	
Hong Kong				
EBV positive	82	18	100	NA
EBV negative	0	0	0	
Missing	4	0	4	
Total	86	18	104	
Singapore				
EBV positive	9	3	12	NA
EBV negative	0	0	0	
Missing	0	0	0	
Total	9	3	12	
Stanford				
EBV positive	34	2	36	0.006
EBV negative	4	4	8	
Missing	0	0	0	
Total	38	6		
All centers				
EBV positive	280	37	317	$3.9e^{-14}$
EBV negative	30	38	68	
Missing	16	1	17	
Total	326	76	402	

Note: A subsample was analyzed for the neuroendocrine markers CgA (n=42) and Synaptophysin (n=42) which were negative, and Ki-67 index which was high in these cases (mean 65.13±24.8; n=38), indicating that NPC is a high-grade tumor, behaving differently clinically than neuroendocrine carcinomas (usually low-grade).

Supplementary Table 3: Stratification of SSTR2 expression and EBV status by geographic location/different sample sets (Fisher’s Exact Test two-sided).

Covariate	Class	Estimate	Std. Error	z	p
Intercept	-	-3.69	1.47	-2.52	0.0117
EBV	Negative	REFERENCE			
	Positive	2.34	0.58	4.03	5.66E-05
Histology	WHO type I	REFERENCE			
	WHO type II	1.11	0.85	1.32	0.189
	WHO type III	2.43	0.85	2.86	0.00422
T	T1-2	REFERENCE			
	T3-4	-0.37	0.38	-0.97	0.33
N	N0-1	REFERENCE			
	N2-3	1.07	0.67	1.6	0.11
M	M0	REFERENCE			
	M1	0.57	0.83	0.69	0.491
Center	Hong Kong	REFERENCE			
	Innsbruck	1.41	0.78	1.81	0.0708
	London	-0.17	1.15	-0.15	0.882
	Shenzhen	2.58	1.32	1.96	0.0505
	Singapore	-1.51	0.92	-1.63	0.102
	Stanford	2.27	0.86	2.65	0.00817
	Utrecht	1.03	0.74	1.39	0.164
	Yogyakarta	1.08	0.84	1.28	0.2
Sample	Primary	REFERENCE			
	Local recurrence	-0.52	0.57	-0.92	0.359
	Metastasis	0.3	0.73	0.41	0.682
Age	-	0.01	0.02	0.65	0.516
Sex	-	0.47	0.42	1.13	0.259

Supplementary Table 4: Multivariate logistic regression model of association between clinical covariates and dichotomised SSTR2 expression (unadjusted two-sided Wald test).

Patient ID	Stage	SUVmax			Biopsy site	SUVmax	IHC score	Radiopeptide
		T	N	M				
NPC-002	T4N3M1	18.9	16.1	15.1	PNS	18.9	3	DOTA-TATE
NPC-007	T3N3M1	10.4	12.5	7.2	PNS	10.4	3	DOTA-TATE
NPC-008	T2N2M1	8.7	9.9	1.1	PNS	8.7	2	DOTA-TATE
NPC-013	T3N2M1	4.9	7.8	14.9	PNS	4.9	3	DOTA-TATE
NPC-017	T2N2M1	6.4	7.3	12.3	PNS	6.4	3	DOTA-TATE
NPC-019	rT3N0M0	12.2	-	-	PNS	12.2	3	DOTA-TATE
NPC-005	rT0N0M1	-	4.1	1.5	Lung	1.5	0	DOTA-TATE
NPC-006	T3N3M0	4.9	3.4	-	PNS	4.9	1	DOTA-TATE
NPC-012	T1N2M0	5.4	10.0	-	PNS	5.4	0	DOTA-TATE
NPC-015	T3N3M1	4.3	22.1	13.8	Skin	1.4	1	DOTA-TATE
NPC-018	T3N3M1	8.1	6.5	3.7	PNS	8.1	1	DOTA-TATE
NPC-020	T1N3M1	4.0	11.1	12	PNS	4.0	1	DOTA-TATE
NPC-003	T4N2M1	13.4	11.3	15.3	-	-	-	DOTA-TATE
NPC-009	rT4N1M1	2.8	3.2	7.4	-	-	-	DOTA-TATE
NPC-014	T4N3M1	9.4	4.8	10.4	-	-	-	DOTA-TATE

Supplementary Table 5: Clinical characteristics and SSTR2 status of NPC patients undergoing ^{68}Ga-DOTA-peptide PET-CT imaging.

Application	Name	Sequence
PCR	SSTR2	F: CTTTCTTGGCTATGCAGGTGG
		R: GAAGATGCTGGTGAACTGATTG
	SSTR2	F: GCACAAGAGGGTCGAGGAG
		R: CATAGCGGAGGATGACATAAATGAC
	EBER1	F: ACGCTGCCCTAGAGGTTTTG
		R: GCAGAAAGCAGAGTCTGGGA
	EBER1 probe	Fam-AGGACGGTGTCTGTGGTTGT-Tamra
siRNA		
	NFKB1	siRNA 1: AUAUUUGAAGGUAUGGGCCAUCUGC
		siRNA 2: UUAUACACGCCUCUGUCAUUCGUGC
	RelB	siRNA 1: GAGGACAUAUCAGUGGUGUUCAGCA
		siRNA 2: GCGAGGAGCUCUACUUGCUCUGCGA
	p52	siRNA 1: CCCAGGUCUGGAUGGUAUUAUUGAA
		siRNA 2: GAUUUCAAAUUGAACUCCUCCAUUG
	c-Jun	siRNA 1: GAUGGAAACGACCUUCUAU
		siRNA 2: GUCAUGAACCACGUUAACA
	RelA	siRNA 1: CCCUUUACGUCAUCCCUGA
		siRNA 2: GGAGUACCCUGAGGCUAUA
	Bcl3	siRNA 1: UACAUUUGCGCGUUCACGUUGGCGC
		siRNA 2: AGCUGCACCAUGCUAAGGCUGUUGU

F: forward, R: reverse

Supplementary Table 6: Overview of PCR primers and siRNA used in this study

Supplementary Figures:

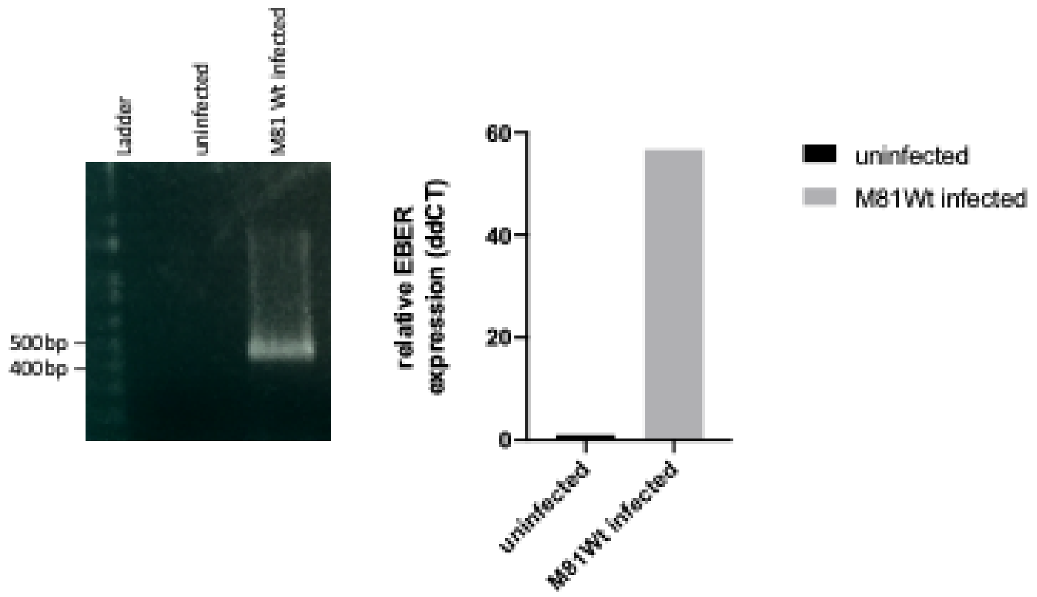

Supplementary Figure 1: RT-PCR analysis of EBV-infected primary epithelial cells using EBER-specific primers and PCR with SSTR2-specific primers confirmed EBV infection and induction of SSTR2 transcription in infected epithelial cells. The figure shows the amplification product (425 bp) after PCR of cells infected or not by the virus. The graph shows the EBER expression levels in infected cells relative to uninfected epithelial cells after normalization with GAPDH signals (ΔΔCT); Replication n=2;

Supplementary results: Cultured primary cells from normal respiratory epithelium were exposed to cell supernatants containing the epitheliotropic virus EBV M81, leading to infection of 5% of the cells, as previously reported[34]. Three days after exposure to the virus, infected cells were detected with an antibody against the nuclear EBNA1 EBV protein. Approximately one third of the EBNA1-positive cells showed intracellular SSTR2 expression (Figure 2A) whereas all cells treated in parallel with supernatants devoid of virus did not show any evidence of SSTR2 expression. RT-PCR analyses with EBER- and SSTR2-specific probes confirmed the infection and the induction of SSTR2 transcription in infected epithelial cells.

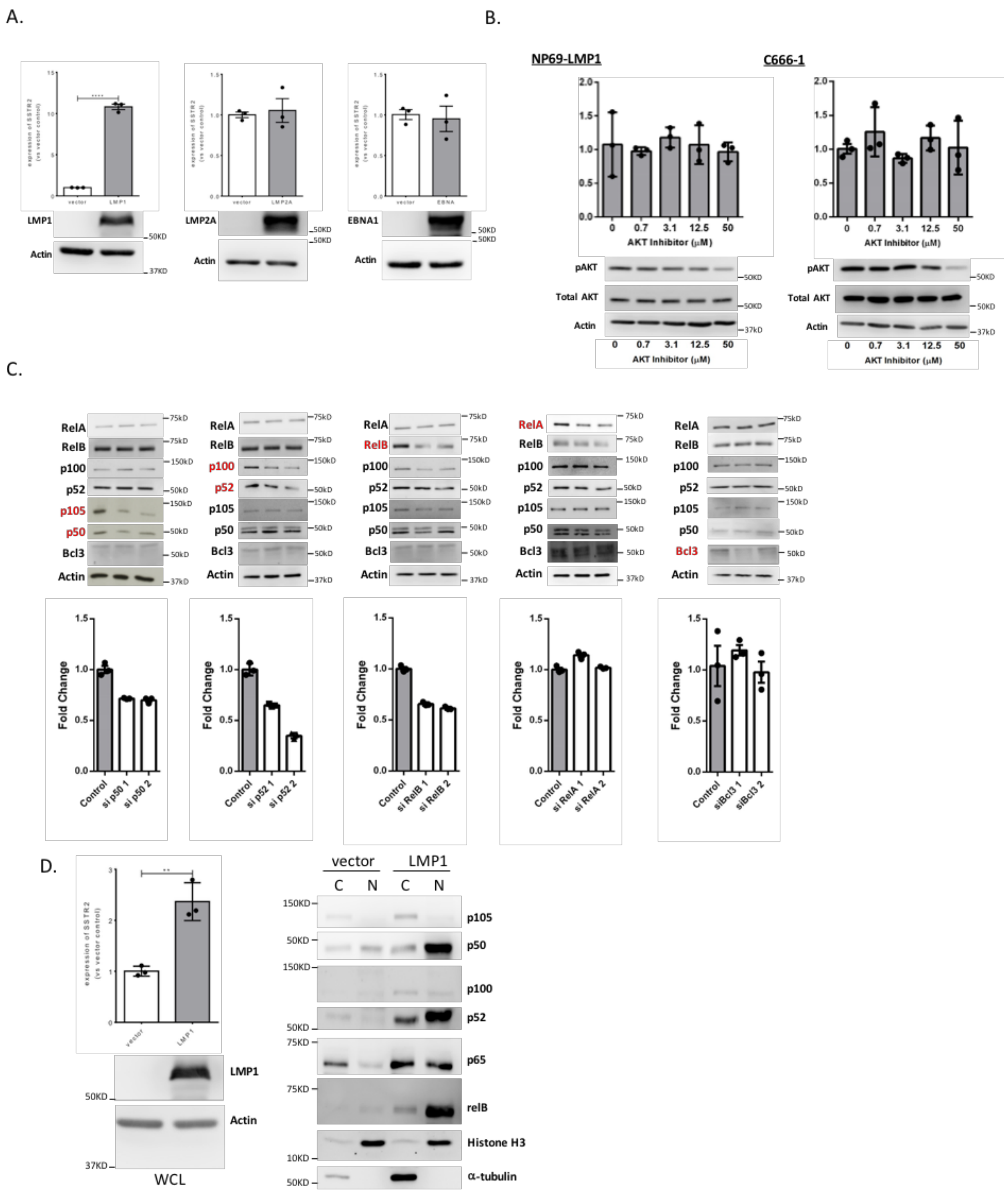

Supplementary Figure 2: LMP1 induces SSTR2 expression in EBV-infected nasopharyngeal epithelial cells. (A) Transient transfection of LMP1, but not other EBV latent genes, EBNA1 and LMP2A induces SSTR2 expression in NP69 nasopharyngeal epithelial cells. (B) In LMP1-expressing NP69 and C666-1 cells, SSTR2 expression was not suppressed by AKT inhibitor treatment. (C) siRNAs mediated knockdown of the subunits of activated NF-κB signal complexes, NFKB1 (p105/p50), NFKB2 (p100/p52), RelB, RelA or BCL3 in C666-1 NPC cells. Significant SSTR2 suppression was shown in NPC cells treated with NFKB1, NFKB2 and RelB siRNAs. (D) LMP1 mediates nuclear accumulation of NF-κB subunits and induces SSTR2 expression.

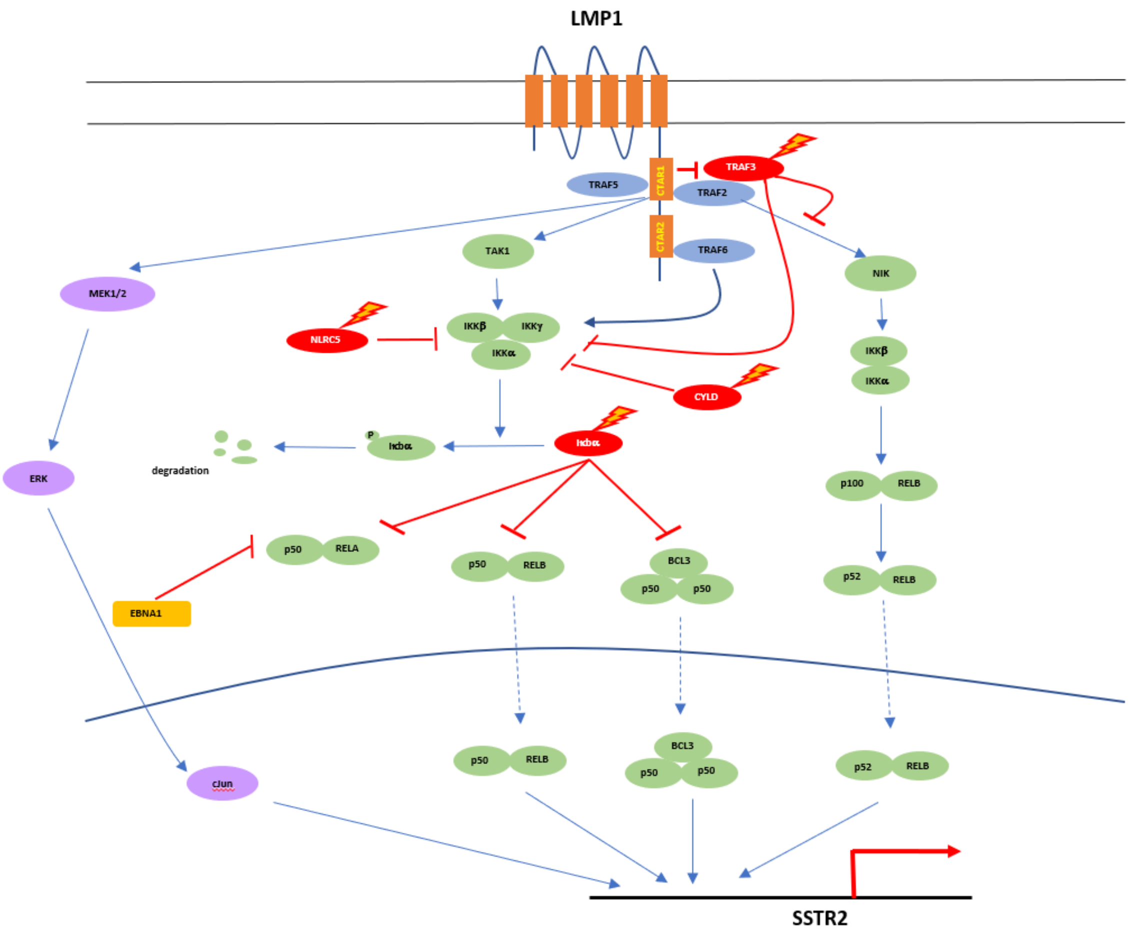

Supplementary Figure 3: Proposed signal transduction pathways downstream of the EBV LMP1, leading to SSTR2 expression.

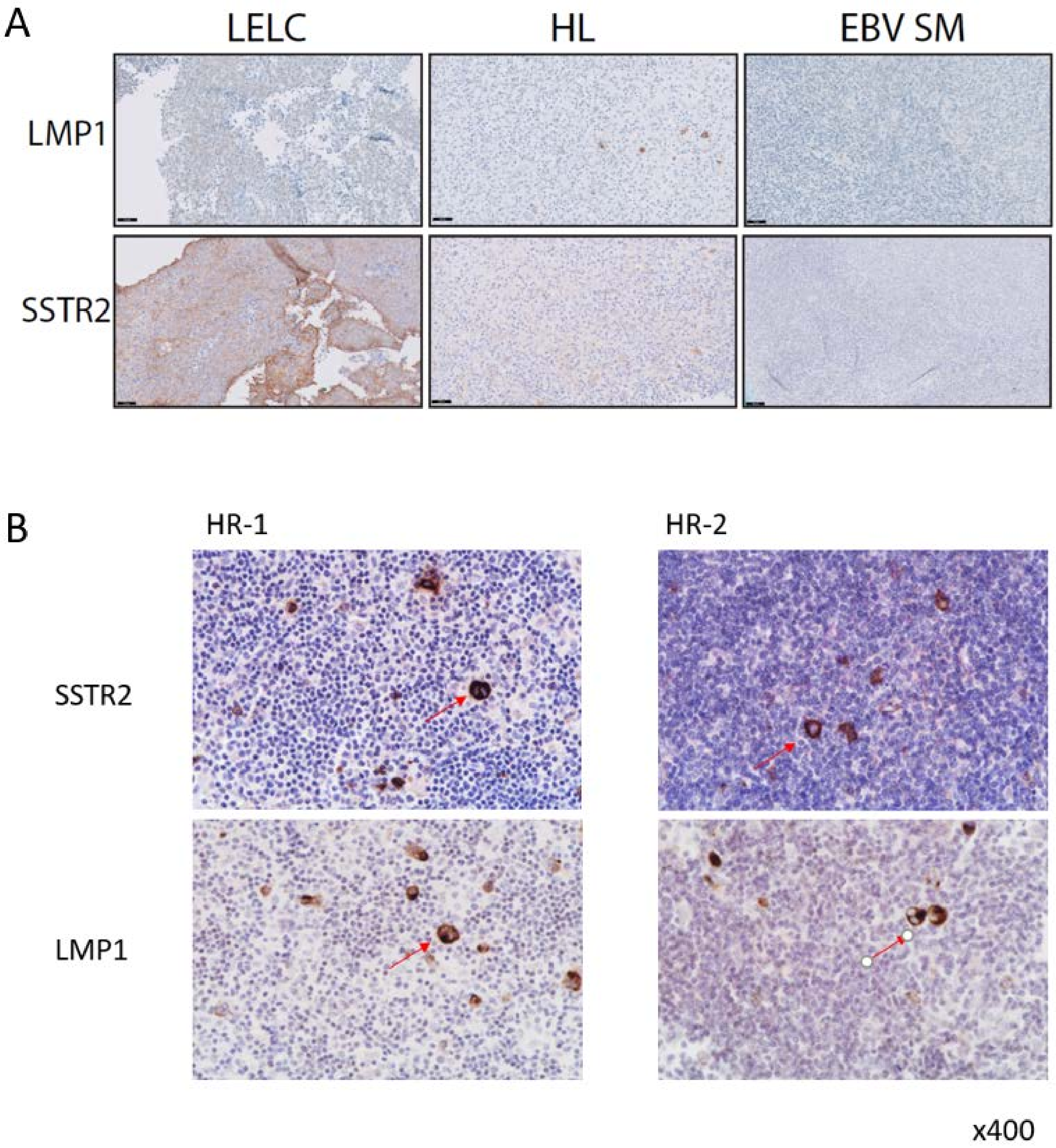

Supplementary Figure 4: A) LMP1 and SSTR2 IHC staining (brown) in EBV related tumors. Lymphoepithelioma-like carcinoma of the lung (LELC) shows strong diffuse staining of SSTR2 and LMP1. In EBV-positive Hodgkin's Lymphoma (HL), LMP1 and SSTR2 staining is shown in the malignant EBV-positive Reed-Sternberg cells, surrounded by inflammatory infiltrate. EBV-associated Smooth Muscle tumor (EBV SM) is absent for both LMP1 and SSTR2. B) High-resolution images (x400) of two cases of Hodgkin's Lymphoma (HL) with strong LMP1 and SSTR2 staining shown in the malignant EBV-positive Reed-Sternberg cells.

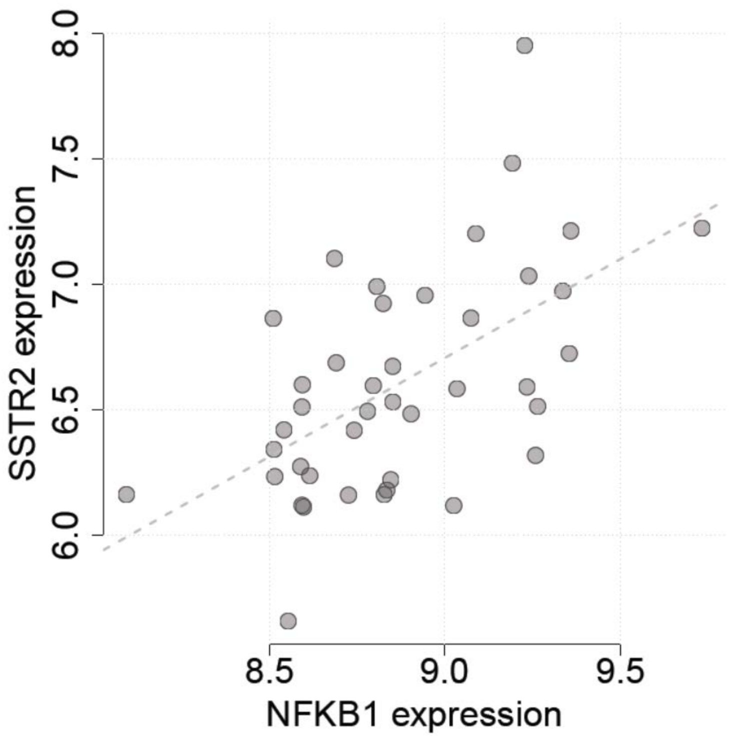

Supplementary Figure 5: A) Positive correlation between SSTR2 and NFkB1 expression (linear regression: b_1=0.78, r^2=0.31, two-sided t-test: p=0.0001), however, no association between NFkB and LMP1 was found in this dataset (data not shown).

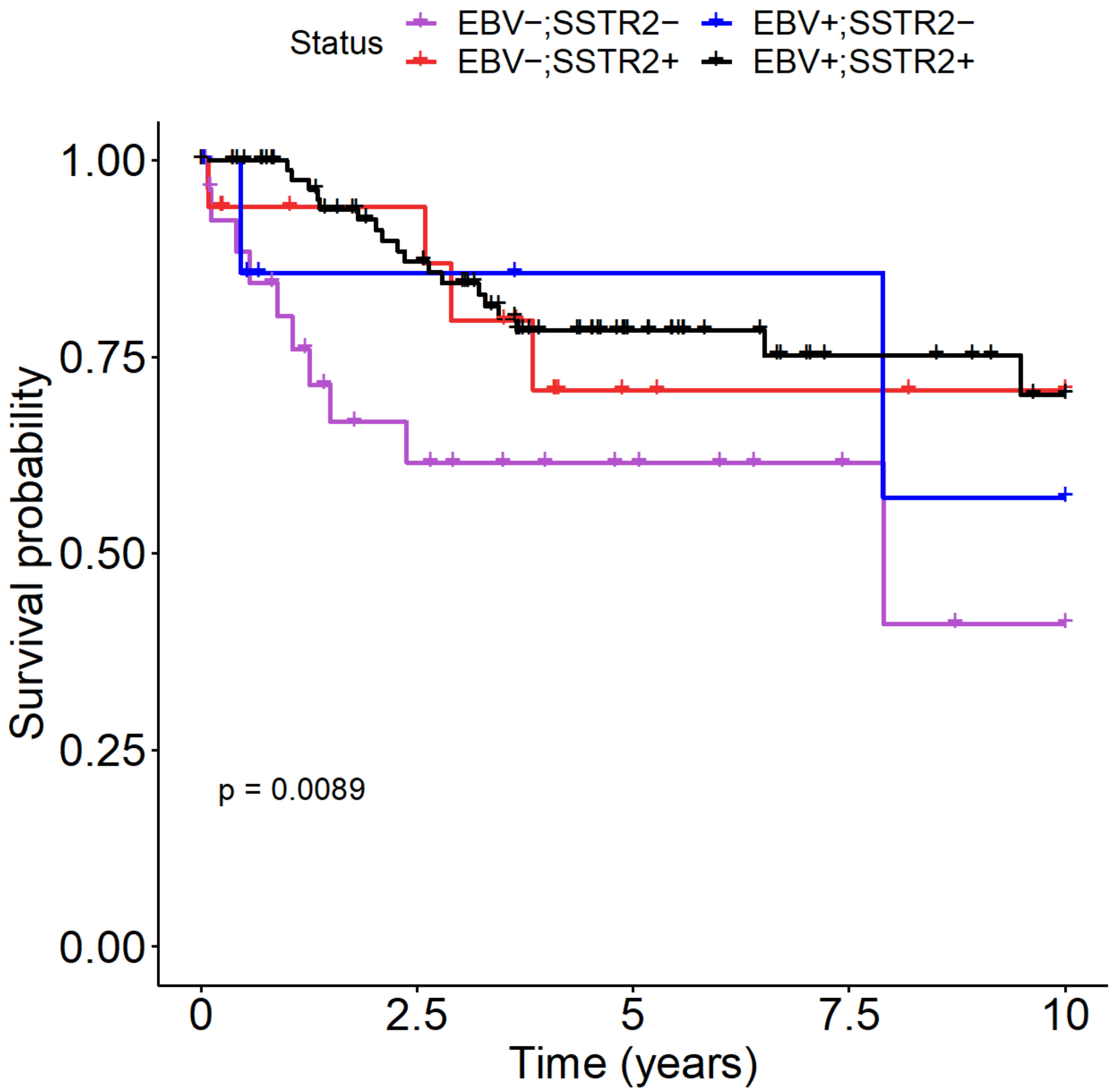

Supplementary Figure 6: Kaplan-Meier curves for European center patients jointly classified by their EBV status and SSTR2 status.

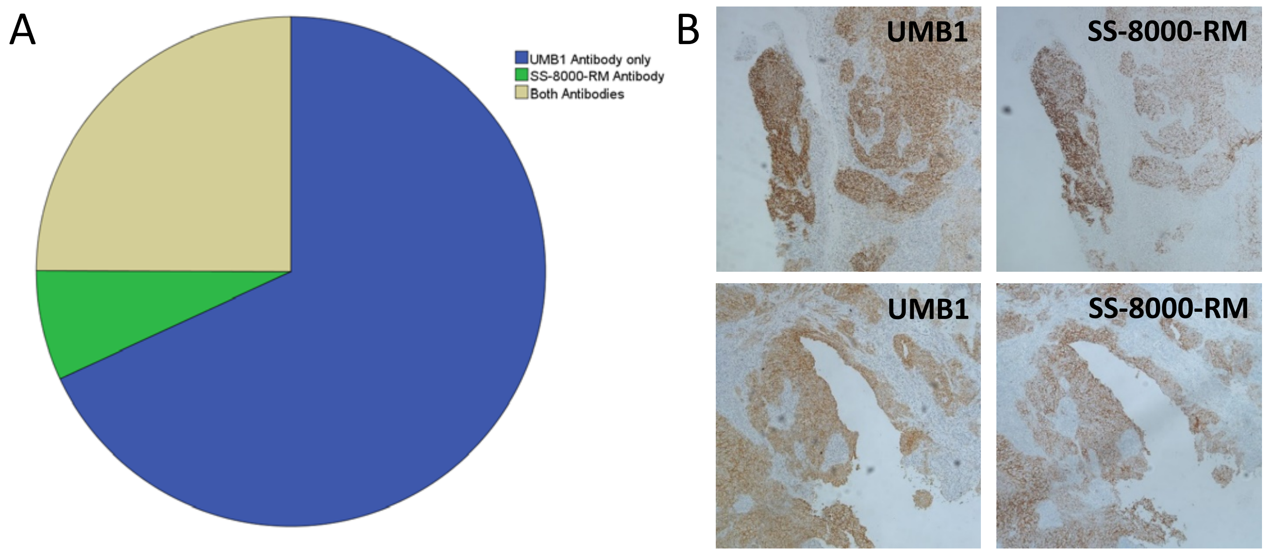

Supplementary Figure 7: Antibodies used for SSTR2A immunohistochemical staining A) Showing a piechart of the antibodies used: UMB-1 antibody alone was used in 273 cases (68.1%) while SS-8009-RM antibody alone was used in 28 cases (7 %). Both antibodies were used in 100 cases (24.9%) with a overall moderate inter-rater reliability (κ=0.49), but a substantial agreement in the 67 cases where tissue samples were available (κ=0.755). Staining in the TMA (n=33) group showed only a slight agreement (κ=0.183) B) Showing two exemplary cases where both antibodies were used in tissue samples (left: UMB1, right: SS-8000-RM). Staining was performed one time with each antibody using positive controls.

Supplementary Notes:

Supplementary Note 1:

In Figure 3 C three of the tumors in the PEN221 group appear to have an accelerated tumor growth. This is associated with a tumor size above 150 mm^3. The first injections of vehicle, Ocreotide and PEN-221 occurred when all three groups had an average tumor size of around 150 mm^3. There were no significant differences in tumor sizes between these groups. Within each group, a few tumors larger than 150mm^3 grew to the humane endpoint quickly, such that none of the treatments, including PEN-221 had any effect on tumor growth or lifespan. When scrutinizing tumors from all three groups, smaller than the 150mm^3 average, at the time of first injection, PEN-221 is the only treatment that slows tumor growth, therefore extending lifespan. To add to this, if you exclude tumors from the data larger than 150 mm^3, at the time of first injection, there is no significant difference in tumor size between groups, adding strength to the observation that PEN-221 slows tumor growth over time increasing survival. Likewise in tumors larger than 150 mm^3, there is no significant difference in tumor size between groups at time of first injection with no effect of PEN-221 on tumor growth over time. Therefore PEN-221 only had an effect on tumor growth if the tumor size at the time of first injection was less than the 150 mm^3 average.

Supplementary Methods:

SSTR2 staining using UMB1 antibody (Abcam). Paraffin-embedded biopsy specimens and TMA sections of 4 µm thickness were cut and processed in an automated immunostainer (Roche Ventana, Tucson, Arizona, USA). Slides were heated to 75°C for 8 min and deparaffinized by an EZ prep solution. Following pretreatment of the samples with EDTA at 95°C for 16 min and subsequent addition of peroxidase inhibitor for 4 min, anti-SSTR2 antibody (rabbit monoclonal UMB1-clone (Abcam, Cambridge UK) was manually applied at 1:250 final dilution diluted in Ventana´s Antibody Diluent on the sections followed by 60 min incubation at room temperature The slides were next incubated with Optiview HQ Universal Linker and Optiview HRP multimer (Ventana Medical Systems) for 8 min or with the Universal Secondary Antibody on Ventana Classic machines. The final steps included application of H_2O_2 and DAB using commercial DAB-containing Ventana kits, and counterstaining with haematoxylin. During each consecutive step of the staining process the slides were rinsed with reaction buffer. Pancreatic tissue was used as a positive control, with liver and lymphoid tissue as negative controls.

SSTR2 staining using rabbit polyclonal antibodies (BioTrend, Cologne Germany). Paraffin-embedded biopsy specimens and TMA sections of 4 µm thickness were cut, heated to 75°C for 8 min and deparaffinized by an EZ prep solution. The next steps were pretreating the samples with EDTA at 100°C for 16 min, addition of peroxidase inhibitor for 4 min and manual application of the primary anti-SSTR2 antibody (rabbit polyclonal antibodies (BioTrend, Cologne Germany, code SS-8000-RM**)** at 1:5 dilution on the sections and incubation for 32 min. The slides were next incubated with Optiview HQ Universal Linker and Optiview HRP multimer (Ventana Medical Systems) for 8 min. The final steps were application of hydrogen peroxide and DAB followed by counterstaining with haematoxylin. During each consecutive step of the staining process the slides were rinsed with reaction buffer. Pancreatic tissue was used as a positive control and liver and lymphoid tissue as negative controls.
