# Supplementary material for: Somatostatin receptor 2 expression in nasopharyngeal cancer is induced by Epstein Barr virus infection: impact on prognosis, imaging and therapy
Source: Nat Commun. 2021 Jan 5;12:117. doi: 10.1038/s41467-020-20308-8 (PMC7785735; doi:10.1038/s41467-020-20308-8)
Supplement: Supplementary file 2 — Reporting Summary [file 41467_2020_20308_MOESM2_ESM.pdf]

nature research

Corresponding author(s): Matt Lechner, Bart Vanhaesebroeck, Valerie J. Lund

Last updated by author(s): Nov 6, 2020

Reporting Summary

Nature Research wishes to improve the reproducibility of the work that we publish. This form provides structure for consistency and transparency in reporting. For further information on Nature Research policies, see Authors & Referees and the Editorial Policy Checklist .

Statistics

For all statistical analyses, confirm that the following items are present in the figure legend, table legend, main text, or Methods section.

n/a	Confirmed	
☐	☒	The exact sample size (*n*) for each experimental group/condition, given as a discrete number and unit of measurement
☐	☒	A statement on whether measurements were taken from distinct samples or whether the same sample was measured repeatedly
☐	☒	The statistical test(s) used AND whether they are one- or two-sided<br>*Only common tests should be described solely by name; describe more complex techniques in the Methods section.*
☐	☒	A description of all covariates tested
☐	☒	A description of any assumptions or corrections, such as tests of normality and adjustment for multiple comparisons
☐	☒	A full description of the statistical parameters including central tendency (e.g. means) or other basic estimates (e.g. regression coefficient) AND variation (e.g. standard deviation) or associated estimates of uncertainty (e.g. confidence intervals)
☐	☒	For null hypothesis testing, the test statistic (e.g. *F*, *t*, *r*) with confidence intervals, effect sizes, degrees of freedom and *P* value noted<br>*Give P values as exact values whenever suitable.*
☒	☐	For Bayesian analysis, information on the choice of priors and Markov chain Monte Carlo settings
☒	☐	For hierarchical and complex designs, identification of the appropriate level for tests and full reporting of outcomes
☒	☐	Estimates of effect sizes (e.g. Cohen's *d*, Pearson's *r*), indicating how they were calculated

Our web collection on statistics for biologists contains articles on many of the points above.

Software and code

Policy information about availability of computer code

Data collection	Data were collected in MS Office Excel (different versions)
Data analysis	Statistical tests and data analysis were performed using IBM SPSS Statistics software (version 24) and R v3.3.1. Differential expression where raw data was available was performed using the R package DESeq2 v1.14.1. Differential expression where only FPKM values were available was performed using the R package limma v.3.30.13. Survival analysis was performed using the R package survival v2.44-1.1. Kaplan meier curves were plotted using the R package survminer v0.4.6. GSEA was performed using the R package clusterProfiler v3.2.14.

For manuscripts utilizing custom algorithms or software that are central to the research but not yet described in published literature, software must be made available to editors/reviewers. We strongly encourage code deposition in a community repository (e.g. GitHub). See the Nature Research guidelines for submitting code & software for further information.

Data

Policy information about availability of data

All manuscripts must include a data availability statement. This statement should provide the following information, where applicable:
- Accession codes, unique identifiers, or web links for publicly available datasets
- A list of figures that have associated raw data
- A description of any restrictions on data availability

The RNA sequencing data from untreated/treated NPC cell lines have been deposited in the GEO database under the accession code GSE160882. The expression array data referenced during the study are available in a public repository from the GEO website under accession GSE12452; https://www.ncbi.nlm.nih.gov/geo/query/acc.cgi?acc=GSE12452. The RNA sequencing data references during the study are available from the GEO website under accession GSE102349; https://www.ncbi.nlm.nih.gov/geo/query/acc.cgi?acc=GSE102349.

Source data underlying Figure 1, Figure 2c-i, Figure 3b-d, Figure 4d, Suppl Tables 1-4, Suppl Figure 2, 6 and 7 are provided as a Source Data file. All the other data supporting the findings of this study are available within the article and its supplementary information files and from the corresponding author upon reasonable request.

Field-specific reporting

Please select the one below that is the best fit for your research. If you are not sure, read the appropriate sections before making your selection.

- [x] Life sciences
- [ ] Behavioural & social sciences
- [ ] Ecological, evolutionary & environmental sciences

For a reference copy of the document with all sections, see nature.com/documents/nr-reporting-summary-flat.pdf

Life sciences study design

All studies must disclose on these points even when the disclosure is negative.

Sample size	In immunhistochemical analysis we aimed for at least 100 samples. By including centers world wide we could multiply this number. For NCT PET trial a small sample size of 15 patients suitable for a pilot observational study and achievable within timeframe of 1 year was calculated.
Data exclusions	Pre-established exclusion criteria: samples/patients other than NPC. In PET trial (NCT03670342) patients < 21 yrs, pregnancy or breast feeding
Replication	Replication of experiments are listed within the main manuscript.
Randomization	One trial arm only
Blinding	Histopathological evaluation was blinded. No formal blinding applicable as not a RCT in immunhistochemical analysis. No blinding in NCT PET trial as a pilot observational study.

Reporting for specific materials, systems and methods

We require information from authors about some types of materials, experimental systems and methods used in many studies. Here, indicate whether each material, system or method listed is relevant to your study. If you are not sure if a list item applies to your research, read the appropriate section before selecting a response.

Materials & experimental systems

n/a	Involved in the study	
[ ]	[x]	Antibodies
[ ]	[x]	Eukaryotic cell lines
[x]	[ ]	Palaeontology
[ ]	[x]	Animals and other organisms
[ ]	[x]	Human research participants
[ ]	[x]	Clinical data

Methods

n/a	Involved in the study	
[x]	[ ]	ChIP-seq
[x]	[ ]	Flow cytometry
[x]	[ ]	MRI-based neuroimaging

Antibodies

Antibodies used

Anti SSTR2 (Abcam, ab134152, UMB1), Anti SSTR2 (Biotrend, SS-800-RM), Anti SSTR2 (Thermo Fischer, 711091, 11HCLC), Synapthophysin (Ventana, 790-4407, SP11), Chromogranin A (Ventana, 760-2519, LK2H10), Ki-67 Antigen (Dako, M7240, MIB-1), LMP1 (Dako, M0897, CS.1-4), EBNA1 (non-commercial, clone 1H4)

Validation

All antibodies other than SSTR antibodies except anti-EBNA1 used were commercially available and have been validated by the manufacturers. Information below were received from manufacturers homepage and documents available there.

Synapthophysin:

PERFORMANCE CHARACTERISTICS 1.Immunoreactivity of CONFIRM anti-Synaptophysin (SP11) was determined by testing formalin fixed, paraffin embedded normal and neoplastic tissues. For normal tissues, results are as follows: adrenal gland (2/3), bone marrow (0/3), brain cerebrum (3/3), brain cerebellum (2/3), breast (0/3), cervix 0/3), colon (1/3), esophagus (0/3), heart (0/3), hypophysis (3/3), intestine (0/3), kidney (0/3), liver (0/3), lung (0/3), mesothelium (0/3), nerve (0/3), ovary (0/3), pancreas (2/3), parathyroid (0/3), prostate (0/3), salivary gland (0/3), skin (0/3), spleen (0/3), stomach (0/3), striated muscle (0/3), testis (0/3), thymus (0/3), thyroid (0/3), tonsil (0/3), and uterus (0/3). For neoplastic tissues, results are as follows: atypical meningioma (0/1), glioblastoma (1/1), ependymoma (0/1), oligodendroglioma (1/1), ovarian serous papillary adenocarcinoma (0/1), ovarian mucous papillary adenocarcinoma (0/1), islet cell carcinoma (1/1), pancreatic adenocarcinoma (0/1), testicular seminoma and embryonal carcinoma (0/2), medullary thyroid carcinoma (1/1), papillary thyroid carcinoma (0/1), intraductal, lobular, and infiltrating breast carcinoma (0/3), diffuse B-cell lymphoma in spleen (0/1), small cell lung carcinoma (1/1), squamous cell lung carcinoma (0/1), lung adenocarcinoma (0/1), esophageal squamous cell and adenocarcinoma (0/2), adenocarcinoma in stomach (0/1), intestinal adenocarcinoma and mesenchymoma (0/2), colorectal adenocarcinoma and mesenchymoma (0/4), hepatocellular carcinoma (0/1), hepatoblastoma (0/1), clear cell carcinoma (0/1), adenocarcinoma in prostate (0/1), transitional cell carcinoma in prostate and bladder (0/2), uterine leiomyoma (0/1), endometrial carcinoma (0/1), uterine clear cell and squamous carcinomas (0/3), embryonal rhabdomyosarcoma (0/1), rectal melanoma (0/1), basal cell carcinoma in skin (0/1), squamous cell carcinoma in skin (0/1), neurofibroma and neuroblastoma (1/2), mesothelioma (0/1), Hodgkin's lymphoma (0/1),

diffuse type lymphoma (0/3), transitional cell carcinoma and leiomyosarcoma in smooth muscle (0/3), osteosarcoma (0/1), and spindle cell rhabdomyosarcoma (0/1). 2.Immunoreactivity of CONFIRM anti-Synaptophysin (SP11) was also evaluated by testing a variety of formalin fixed, paraffin embedded neoplastic neuroendocrine tissues. For neuroendocrine tissue, 288 samples were stained and evaluated for positivity. 129/288 neuroendocrine cases demonstrated positive staining with the antibody. 3.Inter-run reproducibility was determined by staining 5 replicate slides containing the same 3 tissues from duplicate sample types across a dynamic staining range (high expressing tumor, low expressing tumor and normal tissue) over 5 days on a BenchMark XT instrument. 148 of 150 tissues tested scored equivalently. 4.Intra-run reproducibility was determined by staining 14 replicate slides containing the same 3 tissues from duplicate sample types across a dynamic staining range on a BenchMark XT instrument. 83 of 84 tissues tested scored equivalently. 5.Intra-platform reproducibility was determined by staining 5 replicate slides containing the same 3 tissues from duplicate sample types across a dynamic staining range over 3 BenchMark XT instruments. 89 of 90 tissues tested scored equivalently.

1.Wiedenmann B, Franke WW. Identification and localization of synaptophysin, an integral membrane glycoprotein of Mr 38,000 characteristic of presynaptic vesicles. Cell 41(3): 1017-1028, 1985. 2.Navone F, Jahn R, Di Gioia G, Stukenbrok H, Greengard P, De Camilli P. Protein p38: an integral membrane protein specific for small vesicles of neurons and neuroendocrine cells. J Cell Biol 103(6 Pt 1):2511-2527, 1986. 3.Buffa; R, Rindi G, Sessa F, Gini A, Capella C, Jahn R, Navone F, De Camilli P, Solcia E. Synaptophysin immunoreactivity and small clear vesicles in neuroendocrine cells and related tumours. Molecular and Cellular Probes 1(4): 367-381, 1987. 4.Gould VE, Wiedenmann B, Schwechheimer K, Dockhorn-Dworniczak B, Radosevich JA, Moll R, Franke WW. Synaptophysin expression is neuroendocrine neoplasms as determined by immunocytochemistry. Am J Pathol 126 (2): 243-257, 1987. 5.Wiedenmann B, Franke WW, Kuhn C, Moll R, Gould VE. Synaptophysin: a marker protein for neuroendocrine cells and neoplasms. Proc Natl Acad Sci (USA) 83(10): 3500-3504, 1986. 6.Gould VE, Lee I, Wiedenmann B, Mall R, Chejfec G, Franke WW. Synaptophysin: a novel marker for neurons, certain neuroendocrine cells, and their neoplasms. Hum Pathol 17(10): 979-983, 1986. 7.Chejfec G, Falkmer S, Grimelius L, Jacobsson B, Rodensjo M, Wiedenmann B, Franke WW, Lee I, Gould VE. Synaptophysin: A new marker for pancreatic neuroendocrine tumors. Am J Surg Pathol 11(4): 241-247, 1987. 8.Kayser K, Schmid W, Ebert W, Wiedenmann B. Expression of neuroendocrine markers (neuronspecific enolase, synaptophysin and bombesin) in carcinoma of the lung. Pathol Res Pract 183(4): 412-417, 1988. 9.Stefaneanu L, Ryan N, Kovacs K. Immunocytochemical localization of synaptophysin in human hypophyses and pituitary adenomas. Arch Pathol Lab Med 112(8): 801-804, 1988. 10.Wiedenmann B, Kuhn C, Schwechheimer K, Waldherr B, Raue F, Brandeis WE, Kommerell B, Franke WW. Synaptophysin identified in metastases of neuroendocrine tumors by immunocyctochemistry and immunoblotting. Am J Clin Pathol 88(5): 560-569, 1987. 11.Sheehan DC, Hrapchak BB. Theory and Practice of Histotechnology, 2nd Edition. The C.V. Mosby Company, St. Louis, 1980. 12.Roche PC, Hsi ED. Immunohistochemistry-Principles and Advances. Manual of Clinical Laboratory Immunology, 6th edition. (NR Rose Ed.) ASM Press, 2002.

Chromogranin A:

RepeatabilityRepeatability studies for anti-Chromogranin A (LK2H10)antibodywere completed to demonstrate:•Inter-lot reproducibility of the antibody.•Intra-run and Inter-run reproducibility on a BenchMark ULTRA instrument.•Intra-platform reproducibility on the BenchMark GX instrument, BenchMark XT instrument and the BenchMarkULTRA instrument.•Inter-platform reproducibility between the BenchMark GX instrument, BenchMark XT instrument and BenchMarkULTRA instrument.All studies met their acceptance criteria.REFERENCES1.Hendy GN, et al. Chromogranin A. Clin Invest Med. 1995 Feb 18(1): 47-652.Wilson BS, Lloyd RV. Detection of Chromogranin in Neuroendocrine cells with a monoclonal antibody. Am J Clin Pathol 115(3): 458-468, 1984.3.Helman L, et al. Chromogranin A Expression in Normal and Malignant Human Tissues. J Cln Investigations. 1988 Aug (82): 686-690.4.Carson F, Hladik C. Histotechnology: A Self Instructional Text, 3rd edition.Hong Kong: American Society for Clinical Pathology Press; 2009.5.Roche PC, Hsi ED. Immunohistochemistry-Principles and Advances. Manual of Clinical Laboratory Immunology, 6th edition. In: NR Rose, ed. ASM Press; 2002.6.Muñoz, D.G., 1990. Monodendriticneurons: a cell type in the human cerebellar cortex identified by chromogranin A-like immunoreactivity. Brain Res. 528 (2), 335-338.

Ki-67:

1.Cattoretti G, Becker MHG, Key G, Duchrow M, Schlüter C, Galle J, et al. Monoclonal antibodies against recombinant parts of the Ki-67 antigen (MIB 1 and MIB 3) detect proliferating cells in microwave-processed formalin-fixed paraffin sections. J Pathol 1992;168:357-63.2.Gerdes J, Lemke H, Baisch H, Wacker H-H, Schwab U, Stein H. Cell cycle analysis of a cell proliferation-associated human nuclear antigen defined by the monoclonal antibody Ki-67. J Immunol 1984;133:1710-5. 3.Gerdes J, Li L, Schlueter C, Duchrow M, Wohlenberg C, Gerlach C, et al. Immunobiochemical and molecular biologic characterization of the cell proliferation-associated nuclear antigen that is defined by monoclonal antibody Ki-67. Am J Pathol 1991;138:867-73.4.Scholzen T, Gerdes J. The Ki-67 protein: from the known and the unknown [review]. J Cell Physiol 2000;182:311-22.5.Key G, Kubbutat MH, Gerdes J. Assessment of cell proliferation by means of an enzyme-linked immunosorbent assay based on the detection of the Ki-67 protein. J Immunol Methods 1994;177:113-7.6.Key G, Becker MHG, Baron B, Duchrow M, Schlüter C, Flad H-D, et al. New Ki-67-equivalent murine monoclonal antibodies (MIB 1-3) generated against bacterially expressed parts of the Ki-67 cDNA containing three 62 base pair repetitive elements encoding for the Ki-67 epitope. Lab Invest 1993;68:629-36.7.FaratianD, Munro A, Twelves C,BartlettJMS. Membranous and cytoplasmic staining of Ki67 is associated with HER2 and ER status in invasive breast carcinoma.Histopathology 2009:54:254-257.8.Claudio PP, Zamparelli A, Garcia FU, Claudio L, Ammirati G, Farina A, et al. Expression of cell-cycle-regulated proteins pRb2/p130, p107,p27kip1, p53, mdm-2, and Ki-67 (MIB-1) in prostatic gland adenocarcinoma. Clin Cancer Res 2002; 8:1808-15

LMP-1:

References/ Références/ Literatur

1. Pallesen G, Hamilton-Dutoit SJ, Rowe M, Young LS. Expression of Epstein-Barr virus latent gene products in tumour cells of Hodgkin's disease. Lancet 1991;337:320-2.

2. Murray PG, Young LS, Rowe M, Crocker J. Immunohistochemical demonstration of the Epstein-Barr virus-encoded latent membrane protein in paraffin sections of Hodgkin's disease. J Pathol 1992;166:1-5.

3. Engel M, Essop MF, Close P, Hartley P, Pallesen G, Sinclair-Smith C. Improved prognosis of Epstein-Barr virus associated childhood Hodgkin's lymphoma: study of 47 South African cases. J Clin Pathol 2000;53:182-6.

4. Vera-Sempere FJ, Burgos JS, Botella MS, Cordoba J, Gobernado M. Immunohistochemical expression of Epstein-Barr virus-encoded latent membrane protein (LMP-1) in paraffin sections of EBV-associated nasopharyngeal carcinoma in Spanish patients.

Oral Oncol, Eur J Cancer 1996;32B:163-8.
5. Baumforth KRN, Young LS, Flavell KJ, Constandinou C, Murray PG. The Epstein-Barr virus and its association with human cancers [review]. J Clin Pathol: Mod Pathol 1999;52:307-22.
6. Cruchley AT, Williams DM, Niedobitek G, Young LS. Epstein-Barr virus: biology and disease [review]. Oral Diseases 1997;3 Suppl 1: S156-63.
7. Rowe M, Evans HS, Young LS, Hennessy K, Kieff E, Rickinson AB. Monoclonal antibodies to the latent membrane protein of Epstein-Barr virus reveal heterogeneity of the protein and inducible expression in virus-transformed cells. J Gen Virol 1987;68:1575-86.
8. Jiwa NM, Oudejans JJ, Dukers DF, Vos W, Horstman A, van der Valk P, et al. Immunohistochemical demonstration of different latent membrane protein-1 epitopes of Epstein-Barr virus in lymphoproliferative diseases. J Clin Pathol 1995:48:438-42.
9. Leong ASY, Cooper K, Leong FJWM. Manual of diagnostic antibodies for immunohistology. Oxford University Press; 199. p. 162.

EBNA1:
1. Humme, S., et al. The EBV nuclear antigen 1 (EBNA1) enhances B cell immortalization several thousandfold. Proceedings of the National Academy of Sciences of the United States of America 100, 10989-10994 (2003).
2. Grässer, F.A., et al. Monoclonal antibodies directed against the Epstein-Barr virus-encoded nuclear antigen 1 (EBNA1): immunohistologic detection of EBNA1 in the malignant cells of Hodgkin's disease. Blood 84, 3792-3798 (1994).

Eukaryotic cell lines

Policy information about cell lines

Cell line source(s)

NP69, C666-1, C15, C17, C18 and NPC43 cell lines were used in this study. NPC patients as described in multiple publications (Busson, P., et al. Establishment and characterization of three transplantable EBV-containing nasopharyngeal carcinomas. Int J Cancer 42, 599-606 (1988).; Tsao, S.W., et al. Etiological factors of nasopharyngeal carcinoma. Oral Oncol 50, 330-338 (2014).; Lin, W., Yip, Y.L., Jia, L. et al. Establishment and characterization of new tumor xenografts and cancer cell lines from EBV-positive nasopharyngeal carcinoma. Nat Commun 9, 4663 (2018) doi:10.1038/s41467-018-06889-5)

Authentication

Cell lines were authenticated by collaborators (Kwok Wai Lo, George Tsao, Pierre Busson)

Mycoplasma contamination

All cell lines tested negative.

Commonly misidentified lines
(See ICLAC register)

No commonly misidentified cell lines were used in this study

Animals and other organisms

Policy information about studies involving animals; ARRIVE guidelines recommended for reporting animal research

Laboratory animals

6-8 week-old female athymic nude mice with a starting weight of around ~20 g. Mice are kept within Home Office limits of 18 – 22C and 40 – 60% humidity and run on a 12 hour light/dark cycle that runs from 7am to 7pm as decribed in the manuscript.

Wild animals

No wild animals were used in this study.

Field-collected samples

No field-collected samples were used in this study.

Ethics oversight

According to Animals (Scientific Procedures) Act 1986; with project license and personal licenses of scientists involved (B.V. at UCL). The experiment had been approved by the University College London's AWERB (Animal Welfare and Ethical Review Body)

Note that full information on the approval of the study protocol must also be provided in the manuscript.

Human research participants

Policy information about studies involving human research participants

Population characteristics

See above. Demographics provided in supplemental material.

Recruitment

Retrospective analysis of FFPE samples.

Ethics oversight

University College London Research Ethics Committee (UCL REC no. 9609/002)

Note that full information on the approval of the study protocol must also be provided in the manuscript.

Clinical data

Policy information about clinical studies
All manuscripts should comply with the ICMJEguidelines for publication of clinical research and a completedCONSORT checklist must be included with all submissions.

Clinical trial registration

NCT03670342

Study protocol	The study protocol for the NCT03670342 can be accessed on reasonable request.
Data collection	Data for the NCT03670342 were collected in National Cancer Centre, Singapore in 2017-2019
Outcomes	Outcome parameters for the NCT03670342:<br>Primary: Number of patients with high somatostatin receptor density (SUVmax >10)<br>Secondary: Correlation with IHC Score
